# Comparing gene-gene co-expression network approaches for the analysis of cell differentiation and specification on scRNAseq data^☆^

**DOI:** 10.1016/j.csbj.2025.05.040

**Published:** 2025-06-06

**Authors:** Alisa Pavel, Manja Gersholm Grønberg, Line H. Clemmensen

**Affiliations:** a Department of Applied Mathematics and Computer Science, Technical University of Denmark, 2800, Kongens Lyngby, Denmark; b Department of Mathematical Sciences, University of Copenhagen, 2100, Copenhagen, Denmark

**Keywords:** Cell differentiation, Gene-gene co-expression network, Systems biology, scRNAseq

## Abstract

Gene-gene co-expression network analysis has been widely applied to bulk RNA sequencing and microarray data to investigate different phenotypes and compound exposures. Recently, it has also been applied to single cell RNA sequencing data. However, the impact of different network models, data processing pipelines, and analysis strategies on downstream interpretations has not yet been characterized.

Here we study the impact of network models and analysis strategies on the resulting interpretations from analyses of cell differentiation and cell state over time using gene-gene co-expression networks.

Our results suggest that the network modeling choice has less impact on downstream results than the network analysis strategy selected. The largest differences in biological interpretation were observed between the node-based and community-based network analysis methods (strategies). In addition, we observe a difference between single time point and combined time point modeling.

## Highlights

• We investigated gene-gene co-expression network modeling approaches.
• Combined time point modeling performed more stable than single time point modeling.
• Differential gene expression-based methods model cell differentiation the best.
• Network analysis strategy has the strongest impact on the results.

## Graphical abstract

**Figure:**
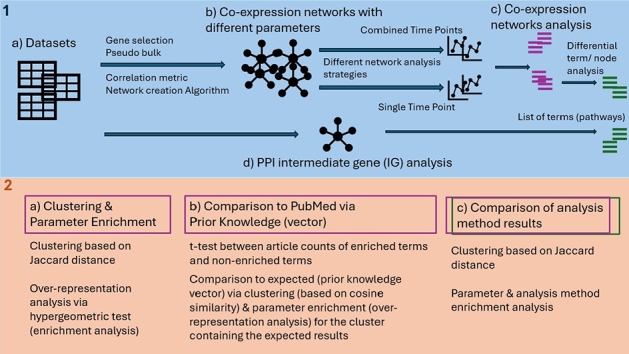

## 1 Introduction

Cells in multicellular organisms can have the ability to differentiate into a multitude of cell types, leading to changes in their morphology and function [1]. Stem cells, and pluripotent stem cells in particular, are of great interest due to their self-renewal capacity as well as the possibility to differentiate into various cells of an organism [2]. Stem cell therapy has many potential clinical applications, ranging from cancer therapy [3] to the treatment of various eye diseases [4]. Cellular differentiation is a complex process that involves gene regulation and expression [5]. Understanding these complex processes and the pathways involved in stem cell differentiation are crucial for manually inducing cell differentiation into specific target cells, minimizing adverse effects, and increasing the differentiation success rate in order to make (wide-spread) clinical application feasible.

Network biology models biological processes and relationships as graphs, enabling the investigation of relationships between entities in a comprehensive manner, rather than focusing solely on individual entities [6]. Protein-Protein Interaction (PPI) networks model relationships between proteins and have been used in drug repositioning, phenotype/compound characterization, and biomarker discovery studies [7], [8], [9], [10], [11], [12], [13], [14], [15]. Gene-gene co-expression networks (GGCN) model gene relationships (co-expression) from omics data and allow a condition's underlying processes to be understood. These types of analyses have found widespread application in understanding diseases, comparing patients, and modeling the impact of chemical exposures [15], [16], [17], [18], [19], [20], [21], [22], [23]. Furthermore, they provide different insights into biological processes compared to traditional gene-centered approaches [23].

Gene-gene co-expression (GGC) is often determined through correlation metrics, however, there are multiple approaches to determine if a correlation is significant or not [15], [24]. For example, (1) weighted correlation network analysis (WGCNA) [25] is a popular method to investigate GGC [17], [16], [26], [27]. WGCNA makes use of the correlation between expression values of gene pairs to build the co-expression network, which is then pruned based on a selected or computed threshold. (2) The ARACNE algorithm [28] is based on mutual information and prunes the resulting network first on a calculated threshold and second on the Data Processing Inequality (DPI) by removing the weakest edge among connected triplets. The DPI states that for an interaction *I* and nodes $n_{1}$, $n_{2}$, and $n_{3}$: $I(n_{1},n_{3})\leq \min[I(n_{1},n_{2});I(n_{2},n_{3})]$ [28]. (3) The context likelihood of relatedness (CLR) algorithm [29] uses mutual information to build the network and network pruning is based on z-scores estimated against a gene's background distribution. (4) The CS-CORE algorithm [30] has been developed to estimate gene-gene co-expression for a specific cell type based on single cell RNA sequencing (scRNAseq) data. (5) The locCSN algorithm [31] is a method developed to estimate cell-specific networks for scRNAseq data while making use of the gene expression distribution.

While gene-gene co-expression network analysis (GGCNA) has been popular for microarray and bulk RNA sequencing (RNAseq) data for some time, it has also recently found application on scRNAseq data [32], [33], [34], [35], [36]. Su et al. (CS-CORE) [30] developed an approach that allows cell type-specific co-expression to be inferred from scRNAseq data, while taking scRNAseq data specific properties, such as sequencing depth variation and measurement errors, into account. The authors showed that their method is able to outperform existing methods when applied to scRNAseq data. Through the profiling of individual cells, scRNAseq data allows the heterogeneity of cell populations to be studied. For example, this allows the study of the cell cycle, cell development, and cell differentiation. On bulk RNAseq data, this may be challenging due to the measurement of expression values across the whole sample population. However, on scRNAseq data, the individual measurement of cells allows the capture of different stages of development/differentiation. This makes it possible to investigate cell development/differentiation in the context of the whole biological system, which can be modeled through a GGCN. Due to the large number of measured cells and their natural variability, gene-gene correlations can be estimated across a single condition from scRNAseq data [30]. In contrast, microarray and bulk RNAseq data are often limited by the number of samples and/or replicates available. Often this results in correlations being computed across conditions to capture changes in gene expression. However, scRNAseq transcriptomics data provide some challenges in comparison to bulk transcriptomics data due to the sample size, the potential number of measured genes, and the sparsity in these measures [37], [38], [39], [40].

Therefore, many current approaches are based on pseudo-bulks, where single cells are combined (often through aggregation of their expression scores) into groups in order to mimic bulk transcriptomics data and minimize data sparsity [35], [41]. Metacells are pseudo-bulks that are based on groups of cells representing specific cell states [41], [42], [43]. HdWGCNA [41] provides WGCNA [25] in a framework adapted to scRNAseq and spatial transcriptomics data, suggesting the use of metacells. This workflow has been used to investigate the drivers behind patient relapse in pediatric T-cell acute lymphoblastic leukemia, uveal melanoma, and intervertebral disc degeneration [34], [32], [33]. The method locCSN [31] suggests the use of metacells, as well as limiting the size of the gene set, to reduce the sparsity and computational complexity. Gene complexity is often reduced (for single cell and bulk transcriptomics data) by selecting the genes with the highest expression, the genes with the highest variable expression, the genes with the highest differential expression, or based on prior knowledge of the condition to be studied, depending on the purpose of the study and the complexity of the data [31], [41], [44], [30], [45], [46], [47].

However, the impact of methodological choices like the gene selection method, pseudo-bulk creation method, pruning algorithm, or correlation metric on the GGCNs and their downstream analysis has not yet been sufficiently investigated. Therefore, we compare different methods for creating pseudo bulks and selecting genes as well as different GGCN generation (pruning) algorithms and metrics. We compare single time point modeling with combined time point modeling in order to determine how this selection impacts the downstream analysis and insights captured through GGCNA, especially to investigate the cell state and cell differentiation (over time). We select cell differentiation as our case study due to its relevance for scRNAseq-based GGCNA, i.e., population heterogeneity and development. First, we study the impact of different GGCN creation strategies on downstream analysis (see section 2.7.1) and investigate if specific network creation strategies bias the downstream interpretations. Second, we compare the biological insights gained from the different GGCN creation strategies to prior knowledge/expected insights (see section 2.7.2) to investigate if specific GGCN creation strategies yield “better” (closer to expected) insights. Last, we compare the biological insights gained from different GGCNA strategies (see section 2.7.3), investigating if different network analysis strategies (including a PPI-based method) yield the same or different insights across different GGCN creation strategies.

## 2 Methods

This section provides an overview of the experimental setup used in our study (section 2.1). Next, it presents the three data sets that were used (section 2.2). Then, it describes the various methods and algorithms tested in our study for constructing the networks (sections 2.3 and 2.4). Section 2.5 describes the different methods for analyzing the constructed networks. Section 2.6 describes the evaluation of the network size. Section 2.7 describes the methodology for comparing the networks in terms of the statistical test and biological interpretation.

### 2.1 Summary of the applied methodology

**Fig. 1:**
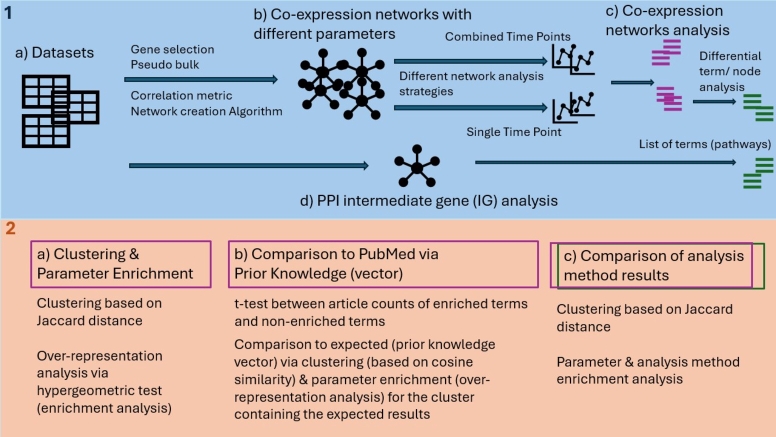
High level visualization of the methods and analysis strategies. First various gene-gene co-expression networks are created using different network creation strategies. This is done for single time point and combined time point modeling (1b). The resulting networks are analyzed with different network analysis strategies, which allows us to annotate the networks with biologically interpretable terms (such as biological pathways and biological processes). In addition, differential time point (term) and differential node centrality analysis is performed on the single time point networks (1c). In parallel to the gene-gene co-expression networks, a PPI-based method (IG) is applied, which makes use of shortest paths on a prior PPI network (1d). Its results are also annotated with biologically interpretable terms. In the second step, the biologically interpretable results are compared to each other. With the help of clustering and over-representation analysis (parameter enrichment), we investigate if specific gene-gene co-expression network creation strategies introduce potential (technical) biases to the downstream analysis (2a). The quality of the biologically interpretable terms is assessed with the help of PubMed and prior knowledge about the investigated datasets (2b). Lastly, the similarity of the biologically interpretable results from the different network analysis strategies is compared (2c).

The applied methodology is separated into two main categories, which are displayed in panels 1 and 2 in Fig. 1. The different parameters and combinations used for the gene-gene co-expression networks are listed in Table 1 and are described in detail in the following sections.

**Table 1:** Parameter and network creation strategy combinations of gene-gene co-expression networks investigated in this study.

Parent Category	Category	# of investigated parameters	Parameters	# of investigated sub-parameters	Sub-parameters	Panel in Fig. 1
	Data Set	3	Rosa et al., Yiangou et al., Close et al.			1a
Data Set	Modeling Method	2	Combined time point, Single time point			1b
Modeling Method	Pseudo Bulk Creation Method	4	leiden clustering (leiden),time point (time),metacells (SEACell),none (only for CS-CORE)	2	with and without 0 expressed genes during collapsing of single cells to meta cells (only for leiden & time)	1b
Modeling Method	Gene Selection Strategy	3	top x most variable,highest expressed,most differentially expressed genes	2	x = [500, 1 000]	1b
Modeling Method	Gene-gene co-expression network	6	ARACNE, CLR,WGCNA, CS-CORE,locCSN, consensus(per time point and combined time point)	3	Pearson correlation,Spearman correlation &mutual information (only for ARACNE,CLR & WGCNA)	1b

**Network creation and analysis (the blue panel in**Fig. 1**)**: The first part of the study consists of the selection of datasets (see section 2.2) (Fig. 1-1a) followed by the computation of GGCNs using different algorithms, correlation metrics, gene selection strategies, and pseudo bulk creation methods. Subsequently, single time point and combined time point networks are created (see sections 2.3-2.4.6) (Fig. 1-1b). The networks are then analyzed with node-based and community-based analysis strategies (shown in purple in Fig. 1). Single time point networks are additionally compared across time points with differential node/term analysis strategies (shown in green in Fig. 1). All results are linked to Reactome pathway and Gene Ontology (GO) terms via gene set enrichment and over-representation analyses (see section 2.5) (Fig. 1-1c). In addition to the analysis of the GGCNs, intermediate gene analysis (IG), which is a prior PPI network-based methodology, is performed for each dataset (see section 2.4.1). This analysis results in a list of Reactome pathway and Gene Ontology terms (shown in green in Fig. 1) (Fig. 1-1d).

**Parameter impact analysis and comparison of analytical results (the orange panel in**Fig. 1**)**: The second part of the study aims to answer the following research questions.

**Fig. 2:**
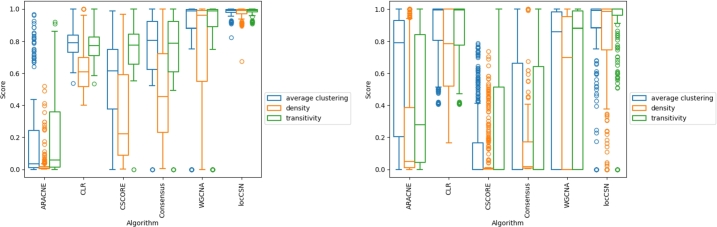
Score: Network density score, average clustering coefficient, and transitivity score distribution across different network creation parameters. Left: Combined time point networks. Right: Single time point networks.

*Which network creation parameters lead to similar enriched Reactome and GO terms?* To investigate this question, the Jaccard distance between the assigned Reactome and GO terms for each created network is computed and clustering is performed on it. Parameter over-representation analysis via a hypergeometric test is performed on each cluster to identify if any of the investigated parameters lead to similar biologically interpretable results (Reactome and GO terms). This analysis is based on the data shown in purple in Fig. 1. The methods are described in section 2.7.1 and the corresponding results are listed in section 3.2 (Fig. 1-2a).

*Which network creation parameters yield the “best” results?* To answer this question, the significant enriched Reactome and GO terms are queried together with prior knowledge about the datasets, such as the cell type, against PubMed [48]. The assumption is that significant enriched terms, which describe the expected processes for the dataset, should return a higher PubMed count than the non significant enriched terms (estimated via a t-test). Parameter over-representation analysis for the network creation parameters is performed via a hypergeometric test after clustering of the t-test results for the cluster containing the expected results (based on the dataset publication). This analysis is based on the data shown in purple in Fig. 1. The corresponding methods are described in section 2.7.2 and the results are listed in section 3.3 (Fig. 1-2b).

*Which of the analysis methods, time point modeling strategies, and network modeling strategies give the most similar results?* To explore this question the Jaccard distance between all Reactome and GO enriched terms is computed and clustering is performed on it. The clusters are investigated for the different network strategies (GGCN vs. PPI network), network creation parameters, modeling strategy (single time point vs. combined time point), and network analysis strategy (node-based vs. community-based). This analysis is based on the data shown in purple and green in Fig. 1. The related methods are described in section 2.7.3 and the corresponding results are listed in section 3.4 (Fig. 1-2c).

### 2.2 Data

We selected three publicly available scRNAseq datasets, each containing more than one time point, in order to investigate cell state, development, or differentiation over time (supplementary Table 1). Two of these datasets (Yiangou et al. and Close et al.) contain pluripotent stem cells. Rosa et al. and Close et al. contain different cell types, which arise over time due to cell differentiation.

#### 2.2.1 Rosa et al.

The normalized count matrix was downloaded from the single cell expression Atlas [49] (download date: 06/2024, ID: E-HCAD-13). The dataset contains fibroblasts, which have been differentiated into dendritic cells at three time points [50].

#### 2.2.2 Yiangou et al.

The normalized count matrix was downloaded from the single cell expression Atlas [49] (download date: 06/2024, ID: E-MTAB-7008). The data contains human pluripotent stem cells under different conditions over two time points [51].

#### 2.2.3 Close et al.

The normalized count matrix was downloaded from the single cell expression Atlas [49] (download date: 06/2024, ID: E-GEOD-93593). The data contains human pluripotent stem cells differentiating into a multitude of (progenitor) cell types across four time points [52].

### 2.3 Processing and pseudo-bulk generation

Since the normalized data matrix was downloaded from the single cell expression Atlas [49], no further processing was performed on the count data.

In order to use GGCN generation algorithms developed for bulk data, pseudo-bulks (clusters/metacells) are computed from the scRNAseq datasets. All pseudo-bulk creation steps are performed with the same parameters for all datasets. We use the following three methods to generate pseudo-bulks:

Method 1) We call this method “*leiden*”. It is based on Leiden clustering [53] (supplementary manuscript section 2.1). The clusters are labeled based on the most frequently occurring time point per cluster as provided by the datasets.

Method 2). This method is denoted as “*time*”. It is based on the time point labels provided in the datasets. For each time point, 10 clusters of 100 samples (cells) are randomly sampled, where samples are allowed to be part of multiple clusters. “Single cell time”, in the results section, refers to non pseudo bulk networks, created from the time point labels (only applicable for CS-CORE [30]).

Method 3) This method is denoted as “*SEACell*”. It creates metacells [42] and each metacell is labeled based on the most frequently occurring time point of its assigned cells (supplementary manuscript section 2.2).

For pseudo-bulk methods 1 and 2, we create pseudo batch expression values by taking the median expression for each gene while a) ignoring zero values per cluster (pseudo-bulk) and b) considering zero values. By both ignoring and considering zeroes, it is possible to evaluate if dropouts impact the downstream interpretation of GGCNA. Pseudo-bulk methods that consider zeroes are denoted by “w0” in the results section. For pseudo-bulk method 3, the returned metacell values are used.

For each of the pseudo-bulk options, we select the top 500 and 1000 genes as input for the gene-gene co-expression network algorithms, where “top” is determined based on the following criteria:

1) The T500var and T1000var datasets take the top 500 and 1000 most variable genes across all the pseudo-batches. This is determined by computing the gene variance using the *pandas.var()* function [54], after the gene expression values have been summarized for each pseudo-batch.

2) The T500sum and T1000sum datasets take the union of the top 500 and 1000 highest expressed genes for each time point.

3) The DEG500 and DEG1000 datasets take the 500 and 1000 most differentially expressed genes. We only consider scores between clusters of different time point annotations. The genes are ranked based on the sum of their adjusted p-values, as returned by the differential expressed genes computation function (Scanpy) between each pair of clusters (supplementary manuscript section 2.3).

We selected 500 and 1000 genes for the network construction a) due to the computational complexity of network analysis (especially path-based methods such as betweenness centrality or community detection [15]) and b) to set similar values for all methods. For example, in scRNAseq analysis pipelines like Scanpy [55], the 2000 most variable genes are set as the default. Differential expression analysis may yield a lower number of genes. For example, the case studies performed in [56] return between 200-300 genes based on a differential variable gene analysis. In particular, the computational complexity of network-based analysis can result in the network size being limited. For example, the authors of locCSN [31] suggest to limit the number of genes to reduce computational complexity.

### 2.4 Gene-gene co-expression network generation

Combined time point GGCNs are computed by providing all pseudo bulks at the same time to the network generation algorithm. Single time point networks are created by providing only pseudo bulks of a specific time point to the network generation algorithm. The total number of resulting networks are listed in supplementary manuscript section 3.

ARACNE [28], CLR [29], CS-CORE [30], locCSN [31], WGCNA [25], and a consensus approach are computed as described in supplementary manuscript sections 3.1 - 3.6. A summary of network creation methods and their combinations is listed in Table 1.

#### 2.4.1 Intermediate gene analysis via PPI network

In addition to the GGCN algorithms listed above, we also include a prior network-based method to investigate cell differentiation. The intermediate gene analysis method (IG) has previously been described in [14]. The method is described in detail in supplementary manuscript section 3.7.

### 2.5 Network comparison

The comparisons conducted in this work focus mainly on evaluating the similarity between the downstream interpretations of the networks. This allows us to investigate how the different GGCN generation methods affect the obtainable biological insights.

#### 2.5.1 Node centralities

Degree (DEG), betweenness (BET) and closeness (CC) centrality for each network are computed and the resulting node centralities are functional enriched via a gene set enrichment analysis (GSEA) [57]. The methods and software are described in detail in supplementary manuscript section 4.1.

#### 2.5.2 Communities

Communities detection is performed on the GGCNs and the results are functionally enriched. The methods and software are described in detail in supplementary manuscript section 4.2.

#### 2.5.3 Differential analysis

For the single time point networks, differential analysis is performed. Differential term analysis is performed for both the node- and community-based results, where only unique terms for a time point are considered. Differential centrality analysis [58], [59], [20] performs a GSEA (as described in section 2.5.1 and supplementary manuscript section 4.1), on the ranked gene list, where genes are ranked by their change in rank (based on node centralities) across time points. The rank change is computed pairwise between all time points for the same network creation strategy and summarized into a mean change value.

### 2.6 Network

In order to investigate network size, we compute network density, the average clustering coefficient, and network transitivity for each network with the NetworkX API [60]. The network density *d* for an undirected graph *G* is defined as d=2m/n(n−1) where *n* is the number of nodes and *m* the number of edges in *G*. The average clustering coefficient is the mean over the local clustering of each node in a graph *G*, where the local clustering is the fraction of existing triangles over possible triangles. The transitivity, *T*, of a graph, *G*, is the fraction of possible triangles in *G* defined as T=3⁎(#triangles/#triads).

### 2.7 Evaluation

#### 2.7.1 Parameter impact

In order to investigate the influence of GGCN parameters, we perform clustering on the previously computed Jaccard distance matrices (see sections 2.5.1 (supplementary manuscript section 4.1) and 2.5.2 (supplementary manuscript section 4.2)). The methods are described in supplementary manuscript section 5.1).

#### 2.7.2 Biological interpretability

To evaluate the quality of the biological insights gained through the different network modeling parameters and analysis methods, we compare the resulting Reactome [61] and GOBP [62], [63] terms to prior knowledge about the datasets with the help of the number of existing PubMed [48] publications. This allows us to investigate if the selection of network creation parameters impacts the quality of results gained from the different GGCNs and their analysis strategies. The methods are described in supplementary manuscript section 5.2.

#### 2.7.3 Comparison of biological insights

Lastly we compare the network analysis methods with respect to the insights they provide. The Jaccard similarity between enriched biological terms (Reactome and GOBP) is computed between all networks and analysis methodologies. Louvain clustering on the similarity graph is performed for all parameters and analysis methods (see sections 2.5.1, 2.5.2 and 2.5.3) as described previously. This method corresponds to panel 2c in Fig. 1.

## 3 Results

### 3.1 The network size is influenced by the choice of gene-gene co-expression algorithm

We observe differences in the size of the network, depending on the network algorithm used (Fig. 2). Since we created networks for each parameter in the gene selection methods with the same nodes, the size of the network refers to the number of selected edges for each algorithm. For the combined time point networks, ARACNE tends to create the sparsest networks, while locCSN and WGCNA create the densest networks (Fig. 2, left). For the single time point networks, locCSN tends to create the densest networks, while CSCORE, ARACNE, and the consensus networks showcase sparser networks (Fig. 2, right). However, the variance across datasets increases in comparison to the combined time point networks, suggesting a wider distribution across the individual networks. The individual dataset distributions are displayed in supplementary Figs. 1-8.

Larger networks have higher computational costs for their analyses, which can make some metrics too costly to compute. Path-based metrics are especially affected by network size because all of the shortest paths in the network need to be computed. Additionally, human interpretability is reduced because it is more difficult to visualize larger/denser networks. For larger networks, this may mean that specific types of analyses cannot be performed due to computational complexity, which reduces the insights that are obtainable from the network modeling of the data. Furthermore, if too few edges are removed in the pruning step, then the network may contain noise (false positive edges), which can introduce noise into the analysis. On the other hand, smaller/less dense networks may be pruned too strictly; this results in important connections (edges) being removed and therefore valuable insights may not be visible. Therefore, it is important to consider network size for the downstream analysis and consider if specific network creation parameters yield smaller or denser networks with respect to their information gain/ loss.

### 3.2 Combined time point modeling, together with community analysis, is the least affected by the choice of gene-gene co-expression network creation algorithm

**Fig. 3:**
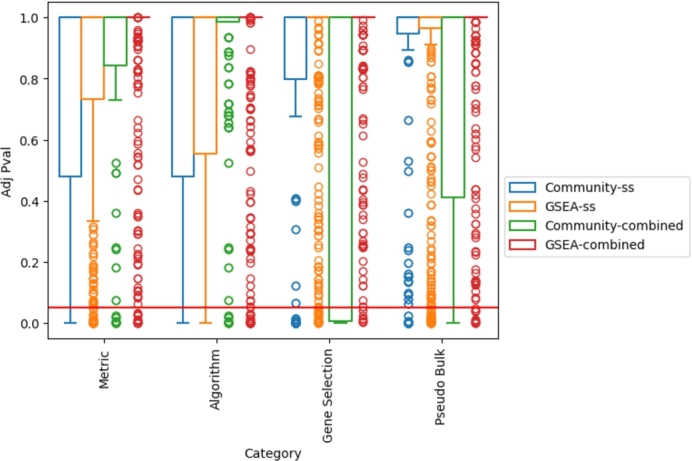
Results of an over-representation analysis on clustered GGCNA results with respect to GGCN creation parameters. Clusters are based on the similarity of the assigned Reactome and GOBP terms of the individual networks. Reactome and GOBP are grouped together, while single time point modeling (ss) and combined time point modeling (combined) are separated. GSEA: (node-based analysis). Community: (community-based network analysis). The red line indicates 0.05.

In order to investigate if specific network creation parameters or analysis strategies influence the biologically interpretable results, clustering of the biologically interpretable results is performed. Parameter over-representation analysis is performed and the results are displayed in Fig. 3, where a statistical over-representation is indicated by the red line (p = 0.05). Parameters that are statistically over-represented in a cluster may indicate parameters that influence the biologically interpretable results of the created networks, which could be caused due to technical or systematic biases of the methods.

It can be seen that the gene selection method has the greatest affect on the similarity of results, followed by the pseudo bulk creation method, especially for community-based analysis (Fig. 3). The effect is especially strong for combined time point analysis in comparison to single time point-based modeling (Fig. 3). This effect can be observed for all three datasets (supplementary figures 10-17). Gene selection, especially based on differentially expressed genes and most variable genes, has the strongest effect on the similarity of GGCNA results (supplementary figure 9). Since gene selection is performed after pseudo bulk creation and on the pseudo bulks, it is indirectly influenced by it; this could explain its impact on the similarity of GGCNA results. In theory, gene selection should especially influence the modeled problem and therefore a downstream effect is to be expected. In contrast, the network creation algorithm and (correlation) metric have the strongest effect on single time point-based modeling (Fig. 3). However, differences between datasets can be observed (supplementary figures 18-25). These results suggest that combined time point modeling (in combination with community-based analysis) seems to be the least affected by technical biases, such as the algorithm and (correlation) metric applied, but rather by the problem modeled (as defined by gene selection). This could be a result of the fact that more data points can be included in combined time point modeling, which can make gene-gene correlations more robust. Additionally, it may reduce the impact of noise in the data, which could be biological or technical, since the effect to be studied (here, cells over time) should have a visible impact on gene expression and gene-gene correlations (given that the effect is contained in the data).

### 3.3 Community analysis provides results most similar to PubMed

**Fig. 4:**
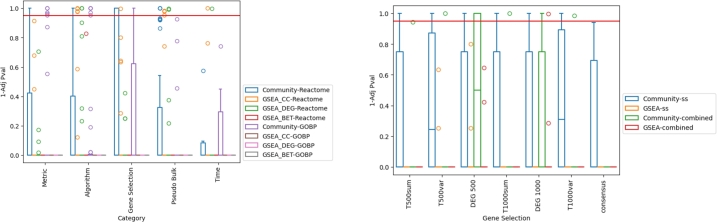
Box plots of 1 minus the adjusted p-value distribution of gene co-expression network cluster enrichment (over-representation) for gene and community-based enrichment on the cluster containing the expected (prior) results. Clusters are based on PubMed scores for the enriched GOBP and Reactome terms. The red horizontal line indicates 0.95. Left: Reactome and GOBP are separated, but combined and single time point modeling are grouped together, displaying the scores across different network creation metric categories. Right: Reactome and GOBP are grouped together, while single time point modeling (ss) and combined time point modeling (combined) are separated, showcasing the results for the individual gene selection methods. GSEA: node-based analysis, based on node centralities; DEG (degree centrality), BET (betweenness centrality), CC (closeness centrality). Community: (community-based network analysis).

In order to evaluate if certain network creation parameters or analysis methods yield “better/more correct” results than others, we compare the biologically interpretable results to PubMed based on prior knowledge about the individual datasets (see section 2.7.2). Fig. 4 showcases the results of an over-representation analysis of the network creation parameter categories after clustering of the PubMed query. A network creation parameter category showcases statistical significant overrepresentation (in accordance with the expected result) if a p-value lower than 0.05 can be achieved (indicated by the red line in Fig. 4). Network analysis strategy, in combination with gene selection method, seems to be the main driver behind the similarity of the interpretable results to expected results, where community-based analysis shows the strongest accordance with the prior knowledge vector (via PubMed). Single time point network modeling does not show any strong difference between the gene selection strategies, however combined time point modeling in combination with a differential expressed gene selection-based strategy showcases over-representation in the prior knowledge vector cluster. However, differences between the datasets can be observed. The results for the individual datasets as well as the other network creation parameter categories are displayed in supplementary figures 26-43.

### 3.4 IG and community analysis provide the most similar results

After investigating the GGCN creation parameters that impact the similarity and quality of the biologically interpretable results, we want to evaluate if different analyses methodologies give the same or different insights, i.e. which strategies may provide a different or the same view on the problem under investigation.

**Fig. 5:**
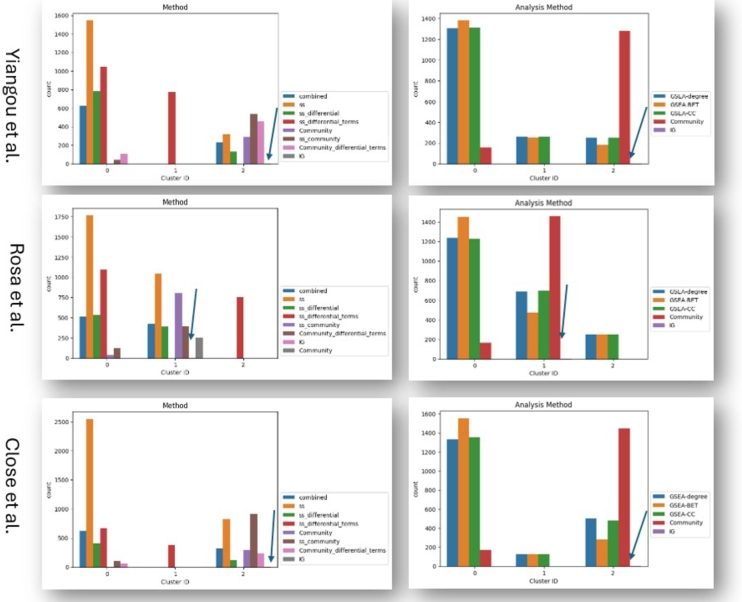
Count plots of parameters falling into different clusters based on the Jaccard distance between resulting Reactome terms for each analysis method. The arrows indicate the location of IG terms (only two terms). Column 1: Combined time point modeling centrality analysis (blue), single time point (ss) modeling centrality analysis (orange), ss differential centrality analysis (green), ss differential term analysis (red), combined modeling community analysis (purple), ss community analysis (brown), ss differential community analysis (pink), intermediate gene analysis (gray). Column 2: GSEA degree centrality analysis (blue), GSEA betweenness centrality analysis (orange), GSEA closeness centrality analysis (green), community analysis (red), intermediate gene analysis (purple).

**Fig. 6:**
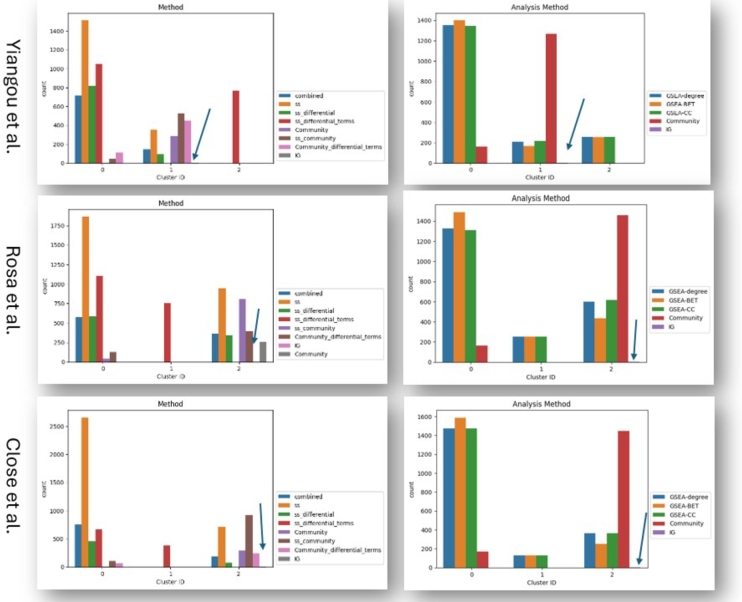
Count plots of parameters falling into different clusters based on the Jaccard distance between resulting GOBP terms for each analysis method. The arrows indicate the location of IG terms (only two terms). Column 1: Combined time point modeling centrality analysis (blue), single time point (ss) modeling centrality analysis (orange), ss differential centrality analysis (green), ss differential term analysis (red), combined modeling community analysis (purple), ss community analysis (brown), ss differential community analysis (pink), intermediate gene analysis (gray). Column 2: GSEA degree centrality analysis (blue), GSEA betweenness centrality analysis (orange), GSEA closeness centrality analysis (green), community analysis (red), intermediate gene analysis (purple).

Fig. 5, Fig. 6 display how analysis methodologies group after clustering based on the similarity of their biologically interpretable results (see section 2.7.3). Across all three datasets, community and GSEA-based analyses are mostly separating, while the two IG results are always grouping together with the majority of the community results (Fig. 5, Fig. 6). These results suggest that GSEA and community-based analysis provide mostly different results with regard to their biological interpretation, while the prior PPI-based approach (IG) gives results in accordance with community-based analysis strategies.

The strong impact of the analysis methodology on the similarity of the results, rather than the GGCN creation parameters (Fig. 5, Fig. 6, and supplementary figures 44-49), suggest that while the different GGCN creation algorithms and parameters give different network (structures) (Fig. 2), their downstream analysis is mostly dependent on the analysis strategy applied. The network creation parameter that impacts the results the most (in combination with community-based analysis) is the gene selection method, which (partly) defines the problem modeled. This suggests that the way the GGCN is created has a lower impact on the downstream insights in comparison to the GGCN analysis strategy. Therefore, researchers need to decide how the networks are analyzed and be aware of the interpretations and insights that can be gained with the different strategies.

## 4 Discussion

GGCNA is a popular method in system biology for understanding different phenotypes or compound exposures [15], [16], [17], [18], [19], [20], [21], [22], [23]. In this study, we investigated how different GGCN creation strategies impact the downstream results when applied to scRNAseq data in order to investigate cell differentiation or cells across multiple time points.

Our results suggest that while different GGCN creation algorithms yield networks with different structures (Fig. 2), the downstream results are mostly influenced by the choice of network analysis method (Fig. 5, Fig. 6). However, we observed that different analysis methods are susceptible to different parameter categories. In addition, we observed differences between single time point and combined time point modeling (Fig. 3). Community-based analysis showed a strong effect to the gene selection method, especially for combined time point modeling. Since gene selection directly affects the problem modeled, this is to be expected and may be seen as a desired effect. For example, differentially expressed genes, or most variable genes (across time points/cell states), focus on the genes changing between the studied conditions, while highly expressed genes may focus more on standard processes (which may be of interest when studying individual cell type networks).

When comparing the results to PubMed with the help of prior knowledge about the datasets being studied, it can be observed that community-based analysis gives insights that are most similar to those expected (Fig. 4). Additionally, differentially expressed gene-based selection shows a strong effect in the combined time point modeling.

Lastly, we compared the similarity of results across different classical network analysis strategies for combined time point networks and single time point networks, also including differential node centrality analysis and differential community analysis. In addition, we included a prior PPI network-based method (IG method). Across all three datasets, a similar pattern can be observed. Most of the community-based results and the IG-based results group together, while the node-based results (GSEA) fall into another group. This suggests that node-based and community-based analysis provides different results (Fig. 5, Fig. 6).

While the initial shortest path computation on the prior PPI network for the IG method is expensive (depending on the PPI network size), it is dataset independent and therefore a one time computation, which can be applied to many different datasets and analyses. In contrast, the GGCNs need to be computed and analyzed for each dataset individually but may be able to capture a broader view than the IG-based method (depending on the dataset and gene selection method, i.e., the problem modeled).

The only method able to separate between time points is (ss_differential_terms), which identified unique terms per time point after a GSEA analysis on single time point networks. The same is not observable for the equivalent strategy on community terms (community_differential_terms) (supplementary figures 44-49).

In general, across all insights, it can be observed that combined time point modeling (especially with community-based analysis) seems to be more robust with regard to network creation parameter impact. This could be as a result of the larger sample size (cells) available when modeling all time points combined. Further, for the usecases studied here (cell differentiation/cells over time), combined time point modeling, in combination with community analysis and differential gene-based gene selection, showcases insights that agree the most with those expected. However, community analysis outperforms node-based analysis for both single time point and combined time point modeling. We believe that this may be due to the fact that in the combined time point modeling (especially for differential gene-based gene selection), gene-gene correlations (or similar) are computed across all gene pairs and time points. This naturally favors genes with expression changes over time (which is the axis studied here). In contrast, single time point modeling focuses on the natural variability between gene pairs of a single time point and therefore it may be difficult to capture gene expression changes over time. Community analysis in general may be more robust due to the consideration of genes as part of a larger system rather than as individual entities.

In summary, our results suggest that the choice of GGCNA strategy has the strongest influence on the downstream results, in contrast to the GGCN creation parameters, where community-based analysis seems to be more robust and provides insights more in accordance with external knowledge. Furthermore, for studies focusing on cell development over time, a combined time point-based modeling is suggested, which will a) provide a higher number of samples (pseudo-bulks) for correlation estimation and b) reduce the number of GGCNs to be computed (since only one network is computed instead of one network for each time point). Since the IG method provides results similar to community-based analysis, this may also be a suitable option. However, due to its initial high computation effort, this method may only be suitable if multiple datasets/studies will be conducted (where the same prior PPI network can be used) or the shortest paths are already pre-computed. While our study focuses only on cell differentiation/cell state over time, we believe that the insights could also be applied to other types of studies relying on scRNAseq data, especially studies investigating changes (over time or between conditions), such as (compound) exposure or phenotypic studies. Future work should therefore further explore those findings, together with novel methods that should be developed or could be adjusted to GGCNA (e.g. [64]), across different application domains and dataset distributions.

## CRediT authorship contribution statement

**Alisa Pavel:** Writing – review & editing, Writing – original draft, Methodology, Investigation, Formal analysis, Data curation, Conceptualization. **Manja Gersholm Grønberg:** Writing – review & editing, Methodology, Conceptualization. **Line H. Clemmensen:** Writing – review & editing, Supervision, Project administration, Funding acquisition, Conceptualization.

## Declaration of Competing Interest

None.

## Appendix A Supplementary material

MMC Supplementary Methods and Figures. MMC
