# Supplementary material for: Comparing gene-gene co-expression network approaches for the analysis of cell differentiation and specification on scRNAseq data
Source: Comput Struct Biotechnol J. 2025 Jun 6;27:2747–56. doi: 10.1016/j.csbj.2025.05.040 (PMC12266514; doi:10.1016/j.csbj.2025.05.040)
Supplement: MMC — Supplementary Methods and Figures. [file mmc1.pdf]

Comparing Gene-Gene Co-expression Network Approaches for the Analysis of Cell Differentiation and Specification on scRNAseq Data: Supplementary Manuscript

Alisa Pavel[1], Manja Gersholm Grønberg[1], and Line H. Clemmensen[1,2,*]

[1]Department of Applied Mathematics and Computer Science, Technical University of Denmark, 2800 Kongens Lyngby, Denmark
[2]Department of Mathematical Sciences, University of Copenhagen, Copenhagen, Denmark
[*]Corresponding Author

Contents

1 Data **3**

2 Processing and Pseudo-Bulk Generation **5**
2.1 Pseudo-bulk Method 1 5
2.2 Pseudo-bulk Method 3 5
2.3 Differential Expressed Genes 5

3 Co-expression Network Generation **6**
3.1 ARACNE 6
3.2 CLR 6
3.3 CS-CORE 6
3.4 locCSN 7
3.5 WGCNA 7
3.6 Consensus 8
3.7 Intermediate Gene Analysis via PPI Network 9

4 Network Comparison **9**
4.1 Node Centralities 9
4.2 Communities 10

5 Evaluation **10**
5.1 Parameter Impact 10
5.2 Biological Interpretability 11

6 Network Size **13**
6.1 Combined Co-expression Network 13
6.1.1 All Datasets 13
6.1.2 Yiangou et al. 14
6.1.3 Rosa et al. 15
6.1.4 Close et al. 16
6.2 Single Time Point Co-expression Network 17
6.2.1 All Datasets 17
6.2.2 Yiangou et al. 18
6.2.3 Rosa et al. 19
6.2.4 Close et al. 20

7 Influence on Biologically Interpretable Results **21**
7.1 Combined and Single Time Point Networks 21
7.2 Combined Co-expression Network 22
7.2.1 All Datasets 22
7.2.2 Yiangou et al. 24
7.2.3 Rosa et al. 26
7.2.4 Close et al. 28
7.3 Single Time Point Co-expression Network 30
7.3.1 All Datasets 30
7.3.2 Yiangou et al. 32
7.3.3 Rosa et al. 34
7.3.4 Close et al. 36

8 Results vs. PubMed **38**
8.1 Combined and Single Time Point Networks 38
8.2 Combined Co-expression Network 40
8.2.1 All Datasets 40
8.2.2 Yiangou et al. 42
8.2.3 Rosa et al. 44
8.2.4 Close et al. 45
8.3 Single Time Point Co-expression Network 47
8.3.1 All Datasets 47
8.3.2 Yiangou et al. 49
8.4 Rosa et al. 51
8.5 Close et al. 53

9 Similarity of Analysis Methods and their Results **55**
9.1 Yiangou et al. 55
9.2 Rosa et al. 57
9.3 Close et al. 59

1 Data

Data Set	# of Samples	# of Cell Types	Variable name	# of Time Points	Variable name	# of Genes
Yiangou et al.	1012	1 (under different growth conditions)	NA	2	Factor Value sampling time point	28425
Rosa et al.	5906	2	Factor Value cell type	3 (2 time points + not applicable)	Factor Value time	20037
Close et al.	1733	8	Factor Value inferred cell type - ontology labels	4	Factor Value time	29864

Table 1: Description of datasets

2 Processing and Pseudo-Bulk Generation

2.1 Pseudo-bulk Method 1

Leiden clustering is based on the Scanpy API [1].

```
sc.tl.pca(adata, svd_solver="arpack")
sc.pp.neighbors(adata)
sc.tl.draw_graph(adata)
sc.tl.leiden(adata)
```

2.2 Pseudo-bulk Method 3

The SEACells pipeline is run based on its tutorial [1] and principal component analysis (PCA) is performed through the Scanpy API [1].

```
import scanpy as sc
import SEACells
sc.tl.pca(adata, n_comps=50)
n_SEACells = int(np.floor(adata.obs.shape[0] / 75))
build_kernel_on = 'X_pca'
n_waypoint_eigs = 10
model = SEACells.core.SEACells(adata,
                  build_kernel_on=build_kernel_on,
                  n_SEACells=n_SEACells,
                  n_waypoint_eigs=n_waypoint_eigs,

                  convergence_epsilon = 1e-5)
model.construct_kernel_matrix()
M = model.kernel_matrix
model.initialize_archetypes()
model.fit(min_iter=10, max_iter=50)
SEACell_ad = SEACells.core.summarize_by_SEACell(adata,
        SEACells_label='SEACell',
        summarize_layer='X')
```

2.3 Differential Expressed Genes

The differentially expressed genes are computed with the Scanpy API [1], where differentially expressed genes are computed pairwise between each cluster pair of different time points.

```
sc.tl.rank_genes_groups(adata2, method='wilcoxon')
```

[1] https://github.com/dpeerlab/SEACells/blob/main/notebooks/SEACell_computation.ipynb

3 Co-expression Network Generation

Yiangou et al. includes 622 single time point networks and 313 combined time point networks. Rosa et al. includes 937 single time point networks and 313 combined time point networks. Close et al. includes 1123 single time point networks and 313 combined time point networks. Note that for some metrics or analyses, the resulting networks may have a non-suitable structure, such as being disconnected, in which case they are not taken into account for that specific analysis.

3.1 ARACNE

ARACNE [2] is computed using the minet R package [3] based on mutual information (original version), Pearson correlation, and Spearman correlation.

```
mim = build.mim(estimator = "pearson", disc = "none")

c = aracne( mim)

mim = build.mim(estimator = "spearman", disc = "none")

c = aracne( mim)

mim = build.mim(estimator = "mi.empirical", disc = "equalfreq")
c = aracne( mim)
```

3.2 CLR

We compute CLR [4] using the minet R package [3] using mutual information (original version), Pearson correlation, and Spearman correlation.

```
mim = build.mim(estimator = "pearson", disc = "none")
c = clr( mim, skipDiagonal=1 )

mim = build.mim(estimator = "spearman", disc = "none")
c = clr( mim, skipDiagonal=1 )

mim = build.mim(estimator = "mi.empirical", disc = "equalfreq")
c = clr( mim, skipDiagonal=1 )
```

3.3 CS-CORE

CS-CORE [5] is computed using the CS-CORE R package across all genes. The resulting p-values are corrected for multiple testing with the Benjamini-Hochberg method through R's *p.adjust* method from the *stats* package. Edges with an adjusted p-value of less than 0.05 are selected in the final network. This

pipeline follows the CS-CORE tutorial [2].

CS-CORE is the only algorithm included here that has been developed for scRNAseq data and where the authors do not recommend the usage of metacells or pseudo-bulks. Therefore, in addition to the different pseudo-bulk methods, we apply CS-CORE to the scRNAseq data without pseudo-bulks for each time point individually. This method is denoted “single cell time” in the results section (under Pseudo Bulk). The same genes, as selected when applying pseudo-bulk method 2, are selected for these networks.

```
CSCORE_result <- CSCORE(genes = all genes)
```

3.4 locCSN

locCSN is computed with the locCSN Python API [6] following their tutorial [3]. Even though locCSN has been developed for scRNAseq data, the authors suggest the usage of metacells (pseudo-bulks) to improve performance, therefore locCSN is only applied to the pseudo-bulk data.

```
csn = locCSN.csn(dev = True)
```

3.5 WGCNA

WGCNA [7] is run with the pyWGCNA Python API [8]. We modified the WGCNA function to take correlation metrics other than Pearson. Pearson and Spearman correlations are computed with Python's pandas API [9, 10], while mutual information is computed with a custom function. The pipeline of using pseudo-bulks as input to WGCNA follows the pipeline suggested in hdWGCNA [11] on how to apply WGCNA [7] to scRNAseq data.

```
pyWGCNA_5xFAD = PyWGCNA.WGCNA(species="homo sapiens", anndata=adata)
```

```
Mutual Information

#Based on: https://medium.com/latinxinai/computing-mutual-information-
    matrix-with-python-6ced9169bcb1


def joint_entropies(data, nbins=None):
    n_variables = data.shape[-1]
    n_samples = data.shape[0]
    if nbins == None:
        nbins = int((n_samples/5)**.5)
```

[2] https://changsubiostats.github.io/CS-CORE/articles/CSCORE.html

[3] https://xuranw.github.io/locCSN/docs/vignettes.html

```
    histograms2d = np.zeros((n_variables, n_variables, nbins, nbins))
    for i in range(n_variables):
        for j in range(n_variables):
            histograms2d[i,j] = np.histogram2d(data[:,i], data[:,j], bins=nbins)[0]
    probs = histograms2d / len(data) + 1e-100
    joint_entropies = -(probs * np.log2(probs)).sum((2,3))
    return joint_entropies

def mutual_info_matrix(df, nbins=None, normalized=True):
    data = df.to_numpy()
    #normalize expression values
    dfmax, dfmin = data.max(), data.min()

    data = (data - dfmin)/(dfmax - dfmin)
    n_variables = data.shape[-1]
    j_entropies = joint_entropies(data, nbins)
    entropies = j_entropies.diagonal()
    entropies_tile = np.tile(entropies, (n_variables, 1))
    sum_entropies = entropies_tile + entropies_tile.T
    mi_matrix = sum_entropies - j_entropies
    if normalized:
        mi_matrix = mi_matrix * 2 / sum_entropies
    return pd.DataFrame(mi_matrix, index=df.columns, columns=df.columns)
```

3.6 Consensus

In addition to the networks mentioned above, we also create consensus networks across multiple algorithms. Consensus networks are constructed for the combined time point networks and the single time point networks. The consensus networks are created based on a median edge ranking. For binary networks, all edges receive the same rank, while for networks with edge scores, the edges are ranked by their assigned scores. If signed correlations are used to label the edges, absolute values are used for the ranking. Ranks across all metrics and algorithms are combined to estimate the median rank for each edge. Edges are selected from the ranked median edge ranking until the network is connected. This strategy is already used in other consensus gene-gene co-expression network approaches [12].

A consensus network is created for each gene selection method across all algorithms, metrics, and pseudo-bulk creation methods, as well as a combined consensus network. Only genes (nodes) in the intersection of the combined networks are considered.

3.7 Intermediate Gene Analysis via PPI Network

A prior PPI network is retrieved from HIPPIE [13] (download date: 02/2024), and only edges with a score ≥ 0.73 are considered (high confidence edges, as described by HIPPIE).

For each dataset, the start genes are computed based on the earliest time point available and the top 500 most expressed genes are selected. This is done by a) including zero expression values (denoted as *IG T500w0s* under Gene Selection) and b) ignoring zero expression values (denoted as *IG T500* under Gene Selection). The end genese are define as the top 500 most differentially expressed genes between the earliest and latest time points available. Differentially expressed genes are computed with the Scanpy API [1], as described previously. We assume that the start genes describe the characteristics of the earliest time point, while the end genes indicate the genes most affected over time. The intermediate genes (to be identified) are assumed to be genes involved in the processes (differentiation) taking place between the earliest and latest time points.

We identify potential intermediate genes by computing the shortest path between any two genes on the PPI network with the NetworkX API [14]. Intermediate genes are computed by retrieving genes on the shortest path between all gene pairs between the start and end genes. Only the shortest paths of length > 1 (at least one intermediate gene) are considered. The occurrence of each intermediate gene on one of the shortest paths is estimated and compared to the background distribution of all shortest paths on the PPI network via a hypergeometric test. The hypergeometric test is computed with the Scipy python API [15] and corrected for multiple testing (across all intermediate genes) based on the statsmodels API [16] (a Benjamini-Hochberg correction [17]). Only genes with an adjusted p-value ≤ 0.05 are kept as intermediate genes.

These intermediate genes are enriched (over-representation analysis) through the gseapy API with their *enrichr* function [18], where the reference library is set to Reactome 2022 [19] and GOBP 2023 [20, 21]. Reactome and GOBP terms are considered as enriched if their adjusted p-value is ≤ 0.05 (as returned by gseapy (multiple test corrected across the library terms)).

4 Network Comparison

4.1 Node Centralities

Degree (DEG), betweenness (BET), and closeness (CC) centrality for each network are computed with the NetworkX API [14]. Edge weights are considered equal, since only the pruned networks are included in the analysis.

To compare the functional interpretation of the node centralities, gene set enrichment analysis (GSEA) [22] over the ranked genes is performed with the blitzgsea API [23], where the reference library is set to Reactome 2022 [19] and GOBP 2023 [20, 21].

The enriched terms for each network (adjusted p-value ≤ 0.05 as returned by blitzgsea (across the library terms)) are compared based on their Jaccard distance, computed with the Scipy API [15].

```
blitz.gsea(data, blitz.enrichr.get_library("Reactome_2022"))
blitz.gsea(data, blitz.enrichr.get_library("GO_Biological_Process_2023"))
```

4.2 Communities

We perform community detection on the GGCNs with the Leiden community detection algorithm, as provided by the VOLTA API [24]. If communities cannot be computed, for example due to network structure, they are not included in the community comparison. Enrichment of the communities is performed through the gseapy API with the *enrichr* function [18], where the reference library is set to Reactome 2022 [19] and GOBP 2023 [20, 21]. Only communities with at least 10 genes are considered. We compare the enriched terms (adjusted p-value ≤ 0.05 as returned by gseapy (across the library terms)) based on their Jaccard distance, which is computed with the Scipy API [15]. Both enrichment APIs (gseapy and blitzgsea) make use of the EnrichR [25, 26, 27] gene sets as reference libraries.

5 Evaluation

5.1 Parameter Impact

Louvain clustering (with default parameters) [28] is computed with the NetworkX API [14] on the weighted similarity network (whose adjacency matrix is represented by the transformation of the Jaccard distance into the Jaccard similarity).

We use a hypergeometric test performed through the Scipy API [15], to estimate if a GGCN generation parameter is over-represented in a detected cluster. The resulting p-values are corrected for multiple testing (across all networks) with the statsmodels API [16] (based on a Benjamini-Hochberg correction [17]) and their distributions are compared with Seaborn's Python API [29]. The results of this study are reported in manuscript section 3.2 and correspond to Panel 2a in manuscript figure 1.

5.2 Biological Interpretability

Reactome and GOBP terms (Terms 2) are queried against the NCBI E-utilities API [30] in combination with defined terms (Terms 1) based on knowledge about the datasets (e.g., cell type, cell differentiation) as described in their corresponding publications. Terms 1 is consistent across all datasets, but its expected value is adjusted (see prior knowledge vector) based on the description of the dataset. Terms 1 is defined as [”differentiation”, ”human pluripotent stem cell”, ”time”, ”cell fate”, ”fibroblast”, ”dendritic”, ”stem cell”, ”progenitor cell”, ”neural”, ”neuron”] and Terms 2 is either Reactome Pathway or GO terms. The query returns the number of PubMed articles containing each pair of terms (between Terms 1 and Terms 2).

The distributions of PubMed article counts of enriched (statistically significant based on an adjusted p-value of ≤ 0.05) terms (Reactome and GO) against non-enriched terms are compared via a one-sided t-test computed with the Scipy API [15]. The assumption is that enriched terms should have a higher number of associated publications than non-enriched terms if they enrich for relevant terms. The resulting p-values are multiple test corrected with the statsmodels API (for each Terms 1 term across all networks) [16] (based on a Benjamini-Hochberg correction [17]).

In order to compare the results with expected terms, a prior knowledge vector is created for each dataset containing the Terms 1 terms and a theoretical score is assigned, where 0 indicates that a term is expected to be present and 1 indicates that a term is not expected to be present or its relationship to the dataset is unknown. The prior knowledge vectors for each dataset are listed below. The scores are assigned based on the cell type and dataset descriptions of each of the individual datasets and are assumed to provide an indication of how similar the individual GGCN interpretations are to the expected knowledge. However, we do acknowledge that the individual datasets and biological processes are highly complex and the simply defined prior knowledge vector does not fully capture this complexity. Rather, it should be seen as an indication of what could be expected and not the ground truth. This simplification allows us to compare the thousands of individual networks and their network creation parameters computationally on their quality in a standardized manner without the need for expert knowledge about the individual datasets.

Louvain clustering is performed on the cosine similarity computed by the scikit-learn API [31] between the p-value vectors of the networks (a vector consisting of the t-test adjusted p-value for each of the terms in Terms 1). Louvain clustering [28] is computed with the NetworkX API [14] (with default parameters). Parameter over-representation of the network creation parameters for the cluster containing the defined prior knowledge vector is determined through a hypergeometric test. The hypergeometric test and multiple test correction is computed the same way as described previously. This allows us to determine

if specific network creation parameters tend to provide results similar to the expected results. This method corresponds to panel 2b in manuscript figure 1.

```
stats.ttest_ind(enriched, non-enriched,
    alternative="greater", nan_policy="omit",
    equal_var=False)
```

The prior knowledge vector for the Yiangou et al. dataset:

```
differentiation = 0
human pluripotent stem cell = 0
time = 0
cell fate = 0
fiborblast = 1
dendritic = 1
stem cell = 0
progenitor cell = 1
neural = 1
neuron = 1
```

The prior knowledge vector for the Rosa et al. dataset:

```
differentiation = 0
human pluripotent stem cell = 1
time = 0
cell fate = 0
fiborblast = 0
dendritic = 0
stem cell = 1
progenitor cell = 1
neural = 1
neuron = 1
```

The prior knowledge vector for the Close et al. dataset:

```
differentiation = 0
human pluripotent stem cell = 0
time = 0
cell fate = 0
fiborblast = 1
dendritic = 1
stem cell = 0
progenitor cell = 0
neural = 0
neuron = 0
```

6 Network Size

6.1 Combined Co-expression Network

6.1.1 All Datasets

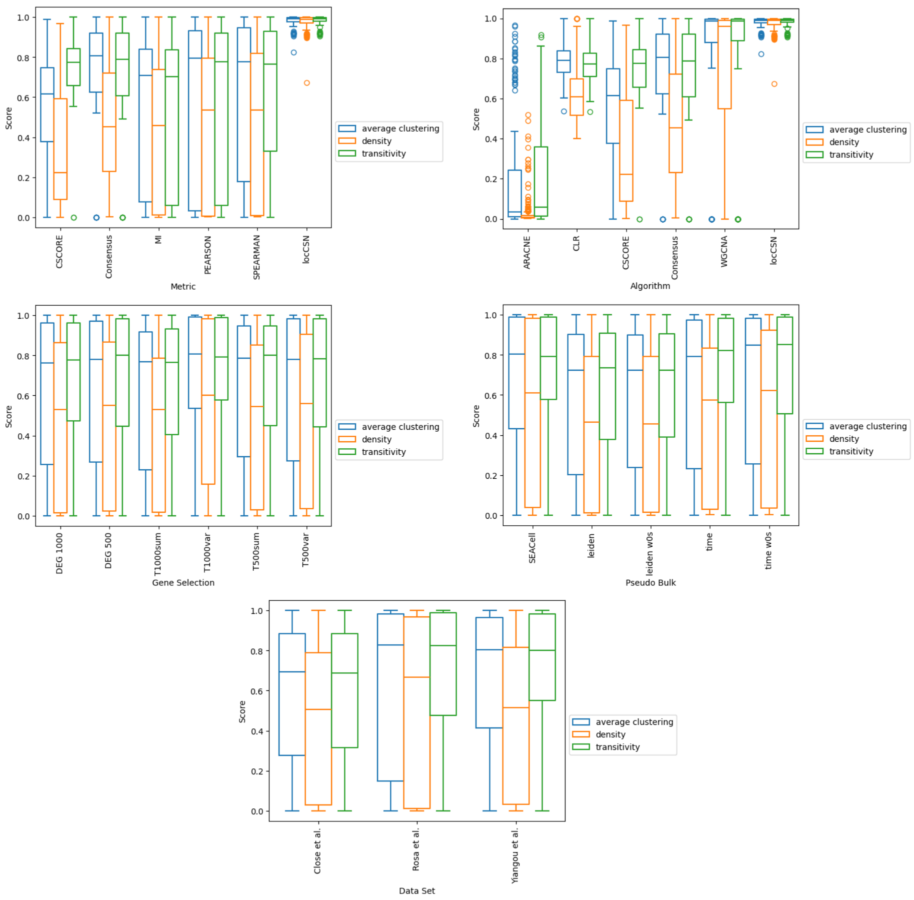

Figure 1: Network density, average clustering coefficient, and transitivity distribution across different network creation parameters.

6.1.2 Yiangou et al.

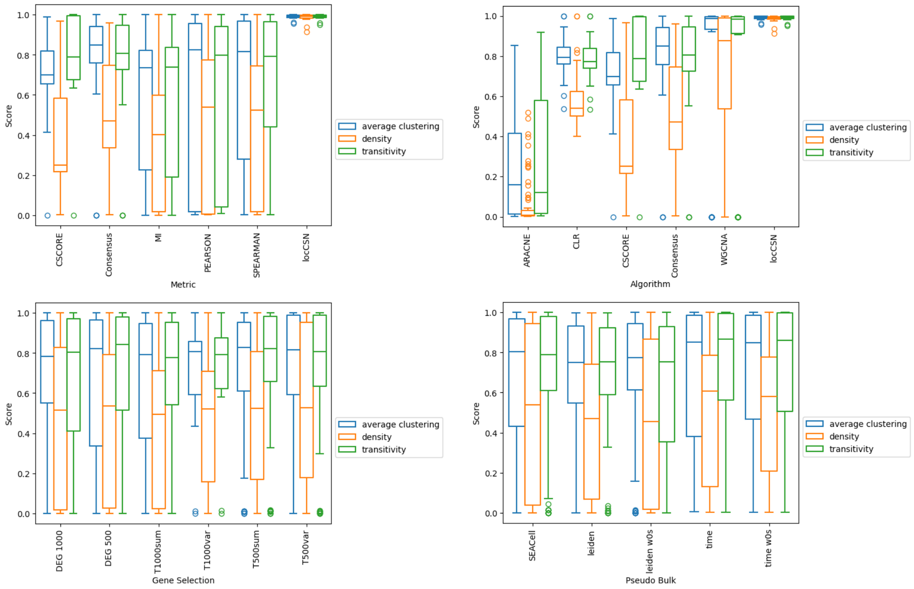

Figure 2: Network density, average clustering coefficient, and transitivity distribution across different network creation parameters.

6.1.3 Rosa et al.

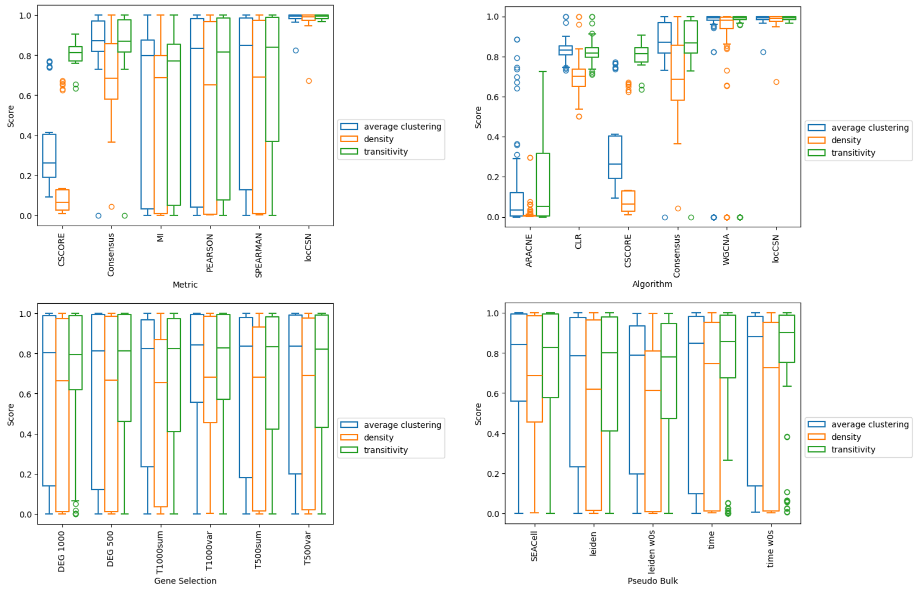

Figure 3: Network density, average clustering coefficient, and transitivity distribution across different network creation parameters.

6.1.4 Close et al.

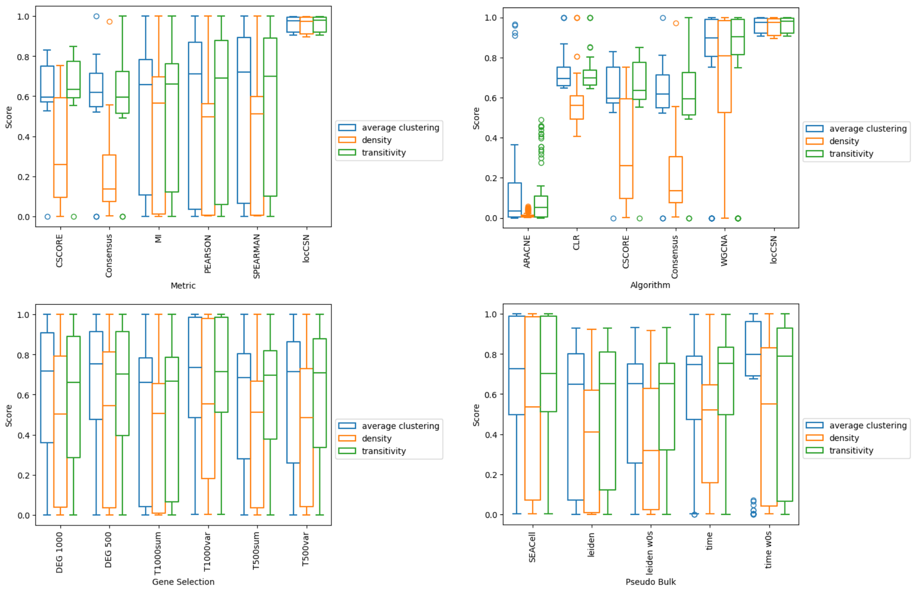

Figure 4: Network density, average clustering coefficient, and transitivity distribution across different network creation parameters.

6.2 Single Time Point Co-expression Network

6.2.1 All Datasets

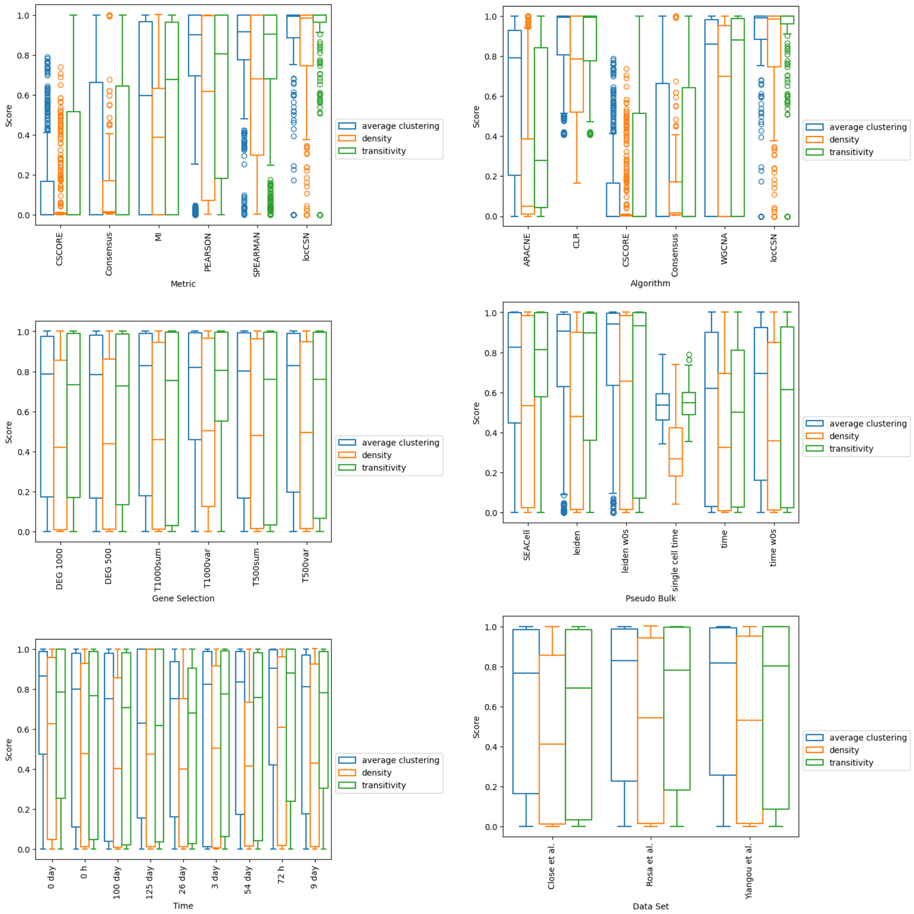

Figure 5: Network density, average clustering coefficient, and transitivity distribution across different network creation parameters.

6.2.2 Yiangou et al.

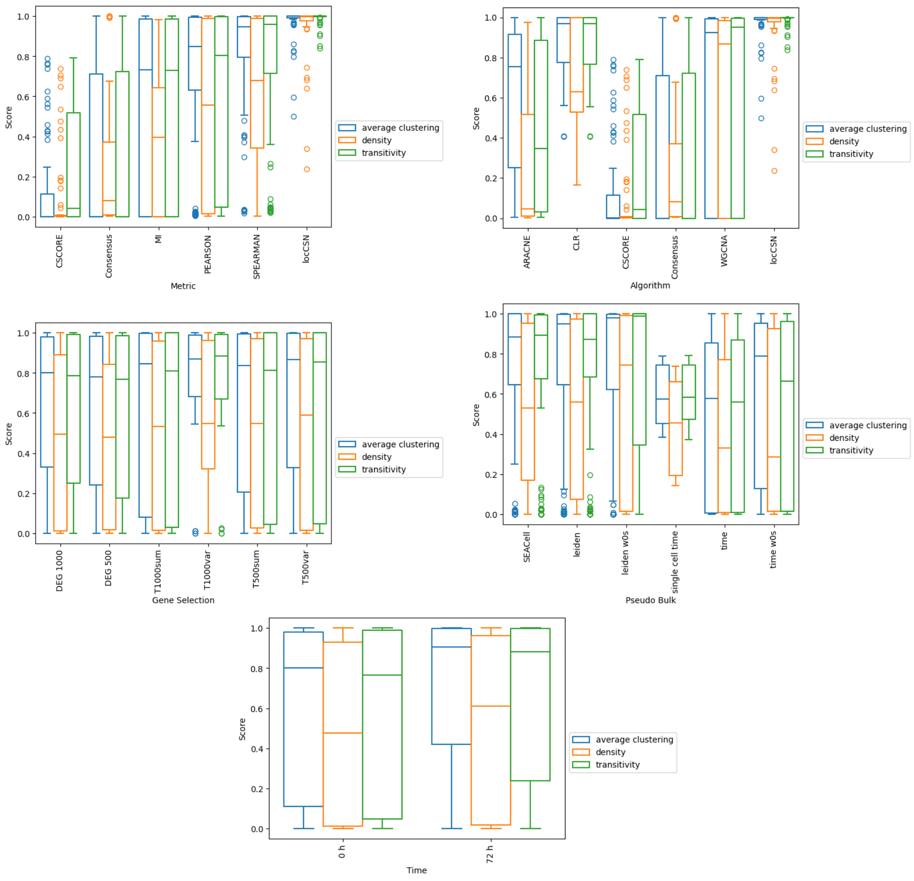

Figure 6: Network density, average clustering coefficient, and transitivity distribution across different network creation parameters.

6.2.3 Rosa et al.

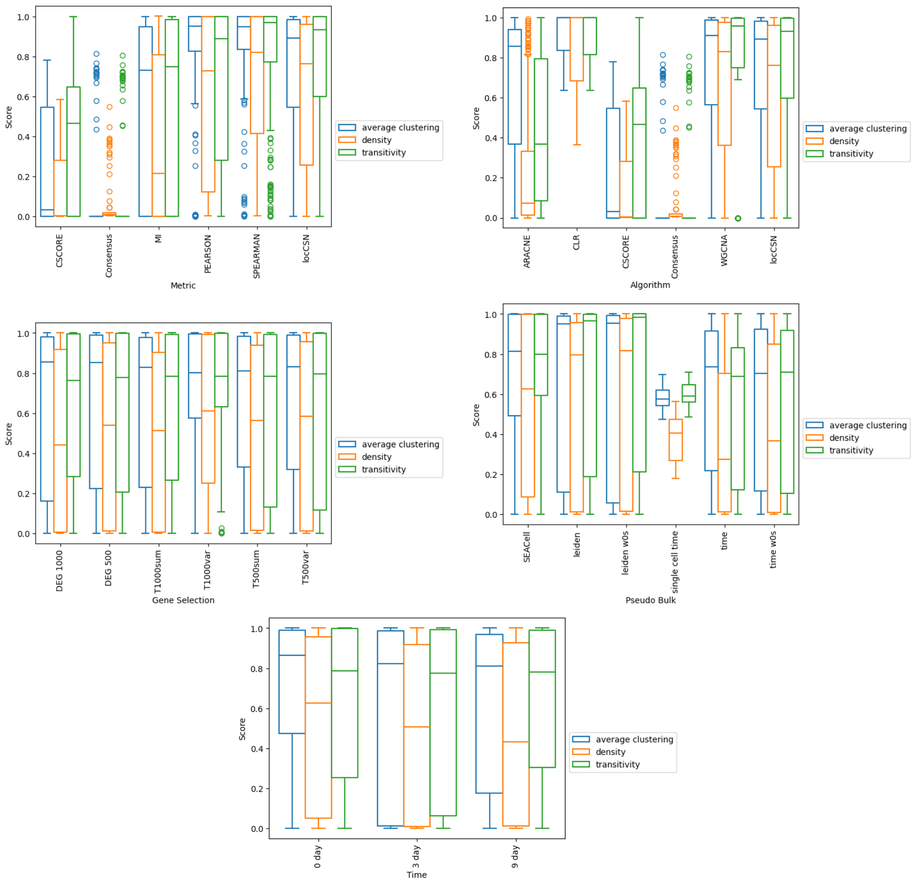

Figure 7: Network density, average clustering coefficient, and transitivity distribution across different network creation parameters.

6.2.4 Close et al.

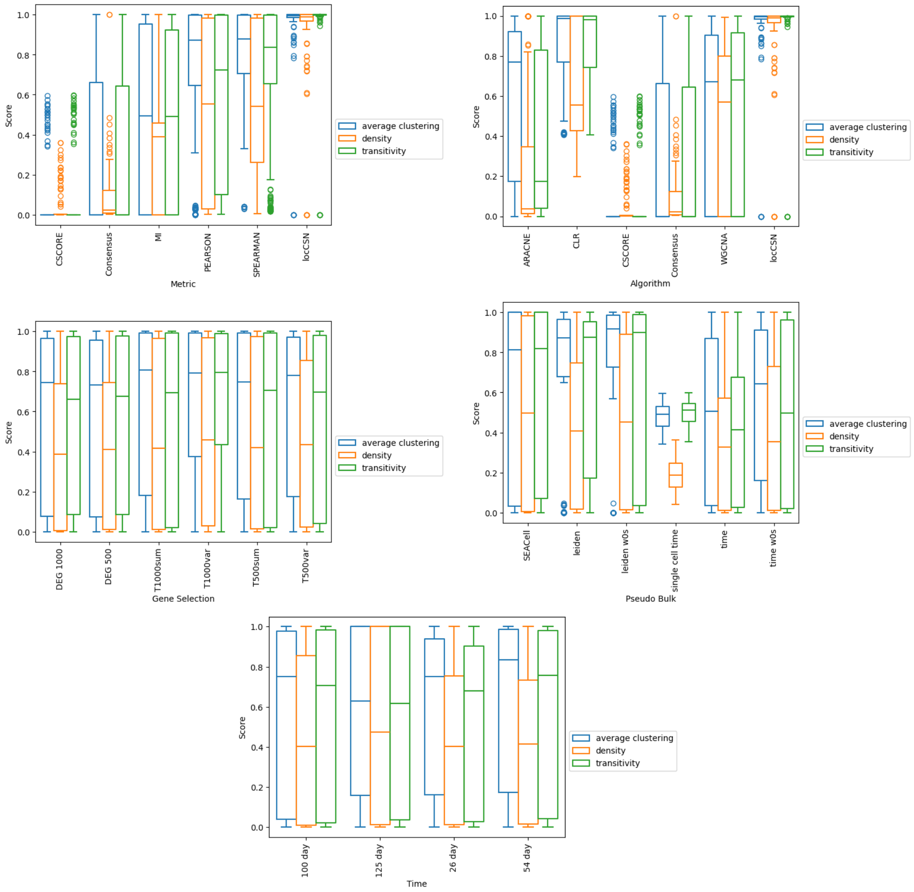

Figure 8: Network density, average clustering coefficient, and transitivity distribution across different network creation parameters.

7 Influence on Biologically Interpretable Results

7.1 Combined and Single Time Point Networks

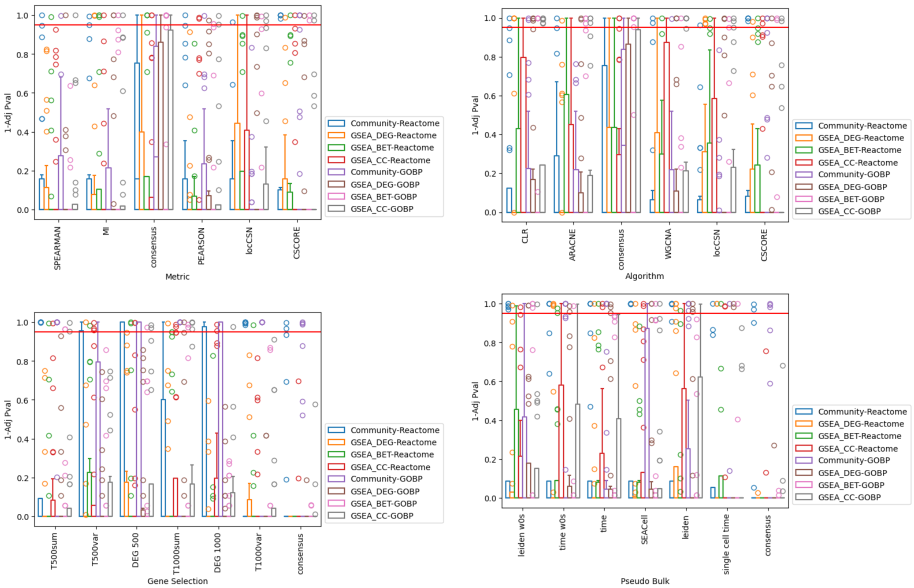

Figure 9: 1- adjusted p-value distribution of gene co-expression network cluster enrichment for gene- and community-based enrichment. Clusters are based on the similarity of assigned Reactome and GOBP terms of the individual networks. The red horizontal line indicates 0.95. GSEA: (node-based analysis, based on node centralities; DEG (degree centrality), BET (betweenness centrality), CC (closeness centrality)). Community: (community-based network analysis).

7.2 Combined Co-expression Network

7.2.1 All Datasets

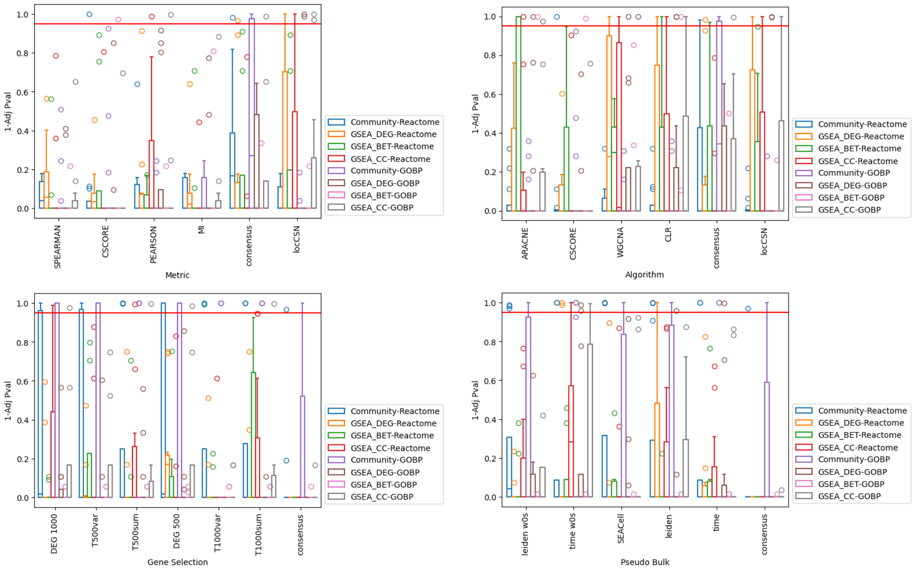

Figure 10: 1- adjusted p-value distribution of gene co-expression network cluster enrichment for gene- and community-based enrichment. Clusters are based on the similarity of assigned Reactome and GOBP terms of the individual networks. The red horizontal line indicates 0.95. GSEA: (node-based analysis, based on node centralities; DEG (degree centrality), BET (betweenness centrality), CC (closeness centrality)). Community: (community-based network analysis).

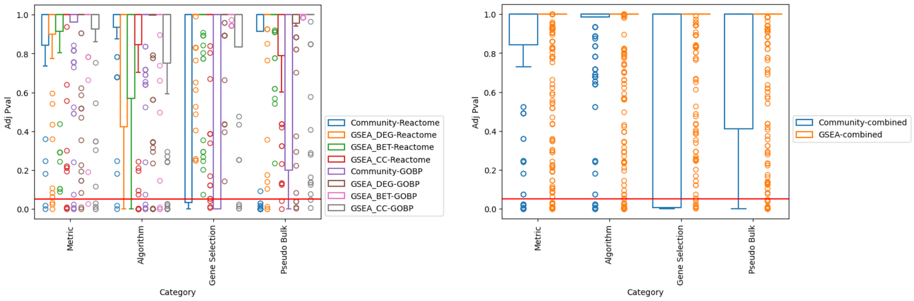

Figure 11: Box plot of parameter categories that are over-represented in clusters grouped by analysis method. If a parameter is over-represented in a cluster, this implies that the parameter influences that clustering, which may suggest a (technical) bias. Clusters are based on the similarity of the assigned Reactome and GOBP terms of the individual networks. Left: Reactome and GOBP are separated, node-based (GSEA) and community-based analyses are also separated. Right: Reactome and GOBP are grouped together, all node-based (GSEA) analyses are also grouped together. GSEA: (node-based analysis, based on node centralities; DEG (degree centrality), BET (betweenness centrality), CC (closeness centrality)). Community: (community-based network analysis).

7.2.2 Yiangou et al.

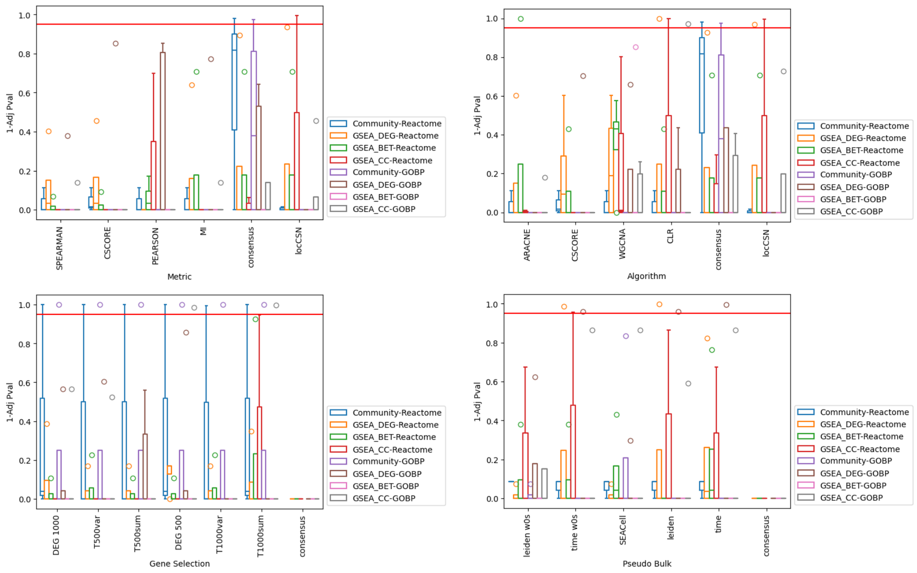

Figure 12: 1- adjusted p-value distribution of gene co-expression network cluster enrichment for gene- and community-based enrichment. Clusters are based on the similarity of assigned Reactome and GOBP terms of the individual networks. The red horizontal line indicates 0.95. GSEA: (node-based analysis, based on node centralities; DEG (degree centrality), BET (betweenness centrality), CC (closeness centrality)). Community: (community-based network analysis).

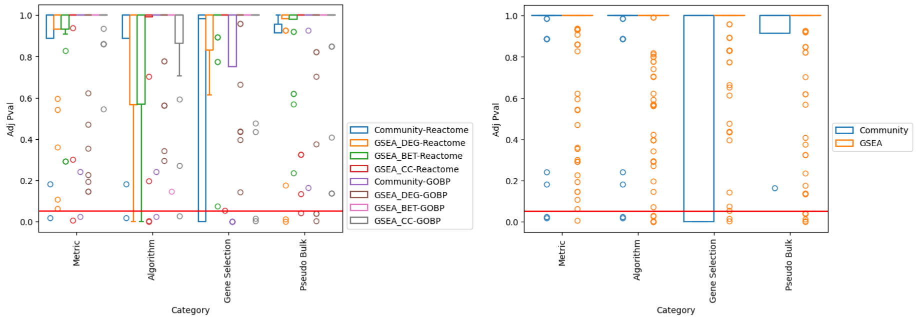

Figure 13: Box plot of parameter categories that are over-represented in clusters grouped by analysis method. If a parameter is over-represented in a cluster, this implies that the parameter influences that clustering, which may suggest a (technical) bias. Clusters are based on the similarity of the assigned Reactome and GOBP terms of the individual networks. Left: Reactome and GOBP are separated, node-based (GSEA) and community-based analyses are also separated. Right: Reactome and GOBP are grouped together, all node-based (GSEA) analyses are also grouped together. GSEA: (node-based analysis, based on node centralities; DEG (degree centrality), BET (betweenness centrality), CC (closeness centrality)). Community: (community-based network analysis).

7.2.3 Rosa et al.

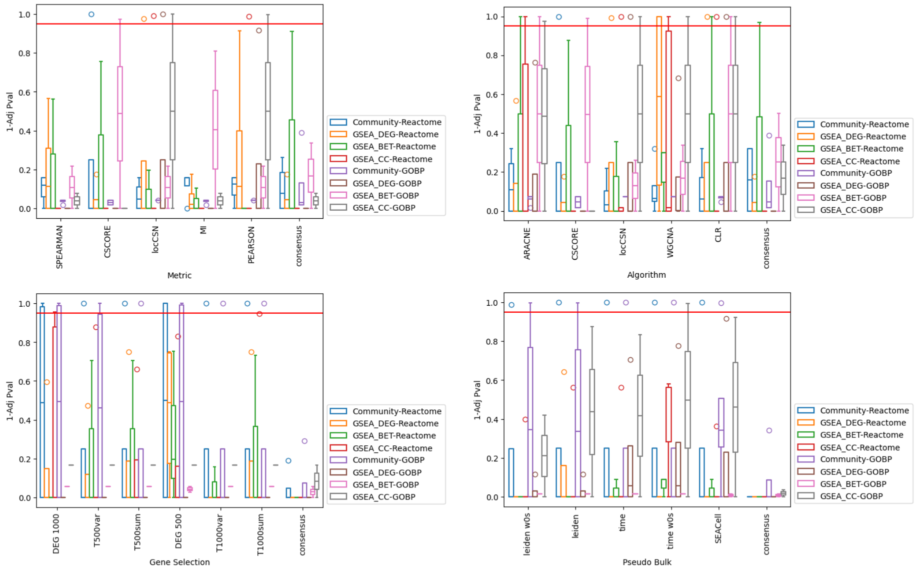

Figure 14: 1- adjusted p-value distribution of gene co-expression network cluster enrichment for gene- and community-based enrichment. Clusters are based on the similarity of assigned Reactome and GOBP terms of the individual networks. The red horizontal line indicates 0.95. GSEA: (node-based analysis, based on node centralities; DEG (degree centrality), BET (betweenness centrality), CC (closeness centrality)). Community: (community-based network analysis).

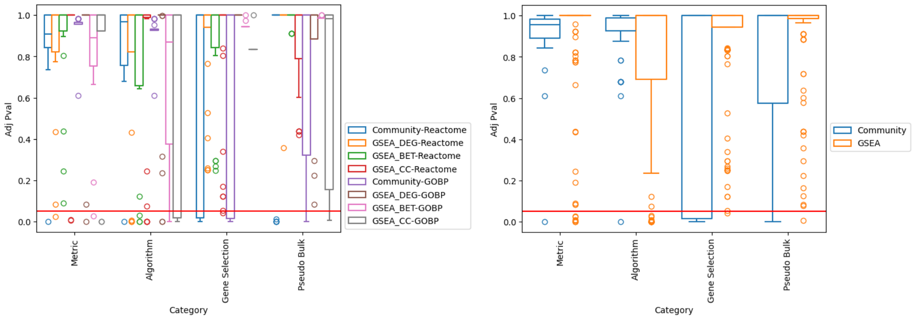

Figure 15: Box plot of parameter categories that are over-represented in clusters grouped by analysis method. If a parameter is over-represented in a cluster, this implies that the parameter influences that clustering, which may suggest a (technical) bias. Clusters are based on the similarity of the assigned Reactome and GOBP terms of the individual networks. Left: Reactome and GOBP are separated, node-based (GSEA) and community-based analyses are also separated. Right: Reactome and GOBP are grouped together, all node-based (GSEA) analyses are also grouped together. GSEA: (node-based analysis, based on node centralities; DEG (degree centrality), BET (betweenness centrality), CC (closeness centrality)). Community: (community-based network analysis).

7.2.4 Close et al.

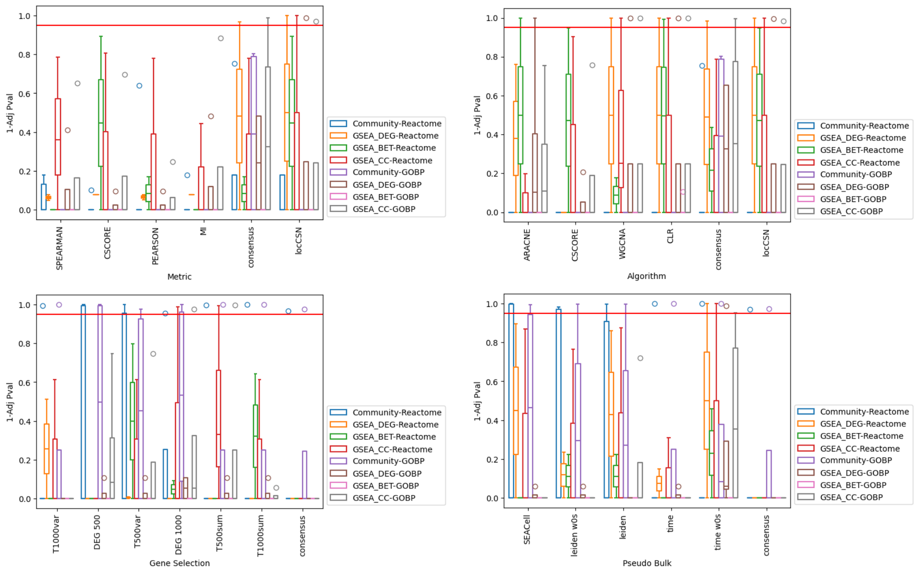

Figure 16: 1- adjusted p-value distribution of gene co-expression network cluster enrichment for gene- and community-based enrichment. Clusters are based on the similarity of assigned Reactome and GOBP terms of the individual networks. The red horizontal line indicates 0.95. GSEA: (node-based analysis, based on node centralities; DEG (degree centrality), BET (betweenness centrality), CC (closeness centrality)). Community: (community-based network analysis).

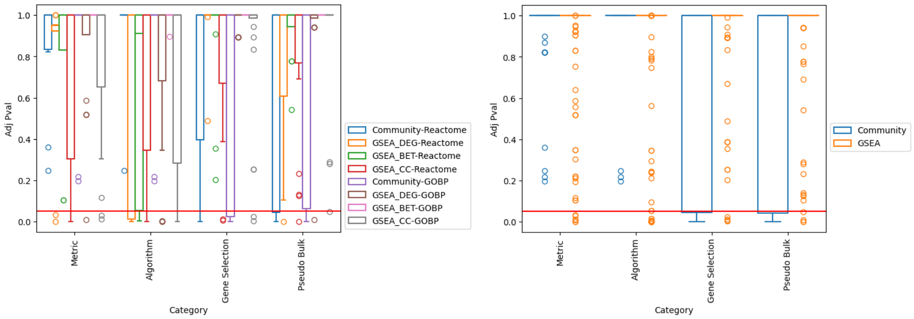

Figure 17: Box plot of parameter categories that are over-represented in clusters grouped by analysis method. If a parameter is over-represented in a cluster, this implies that the parameter influences that clustering, which may suggest a (technical) bias. Clusters are based on the similarity of the assigned Reactome and GOBP terms of the individual networks. Left: Reactome and GOBP are separated, node-based (GSEA) and community-based analyses are also separated. Right: Reactome and GOBP are grouped together, all node-based (GSEA) analyses are also grouped together. GSEA: (node-based analysis, based on node centralities; DEG (degree centrality), BET (betweenness centrality), CC (closeness centrality)). Community: (community-based network analysis).

7.3 Single Time Point Co-expression Network

7.3.1 All Datasets

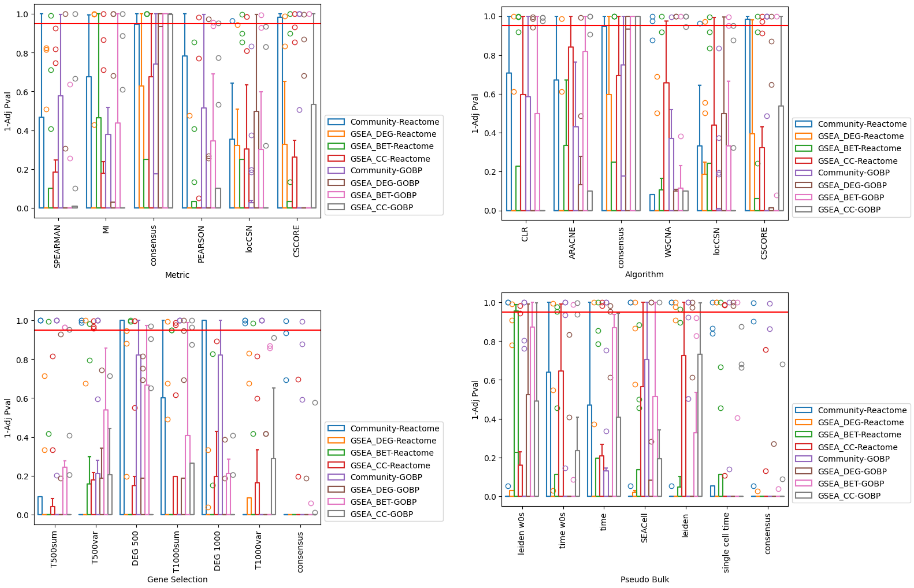

Figure 18: 1- adjusted p-value distribution of gene co-expression network cluster enrichment for gene- and community-based enrichment. Clusters are based on the similarity of the assigned Reactome and GOBP terms of the individual networks. The red horizontal line indicates 0.95. GSEA: (node-based analysis, based on node centralities; DEG (degree centrality), BET (betweenness centrality), CC (closeness centrality)). Community: (community-based network analysis).

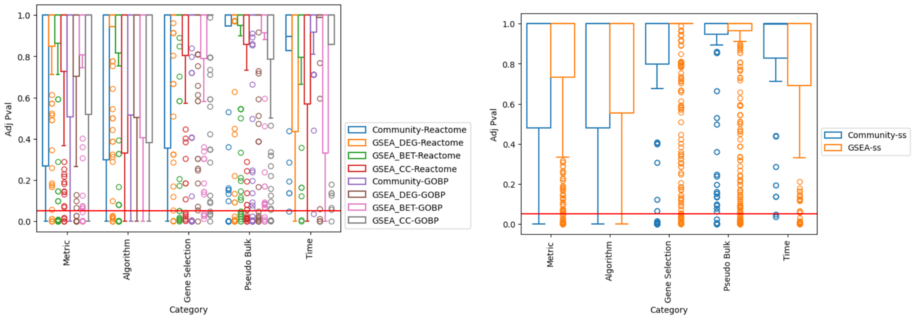

Figure 19: Box plot of parameter categories that are over-represented in clusters grouped by analysis method. If a parameter is over-represented in a cluster, this implies that the parameter influences that clustering, which may suggest a (technical) bias. Clusters are based on the similarity of the assigned Reactome and GOBP terms of the individual networks. Left: Reactome and GOBP are separated, node-based (GSEA) and community-based analyses are also separated. Right: Reactome and GOBP are grouped together, all node-based (GSEA) analyses are also grouped together. GSEA: (node-based analysis, based on node centralities; DEG (degree centrality), BET (betweenness centrality), CC (closeness centrality)). Community: (community-based network analysis).

7.3.2 Yiangou et al.

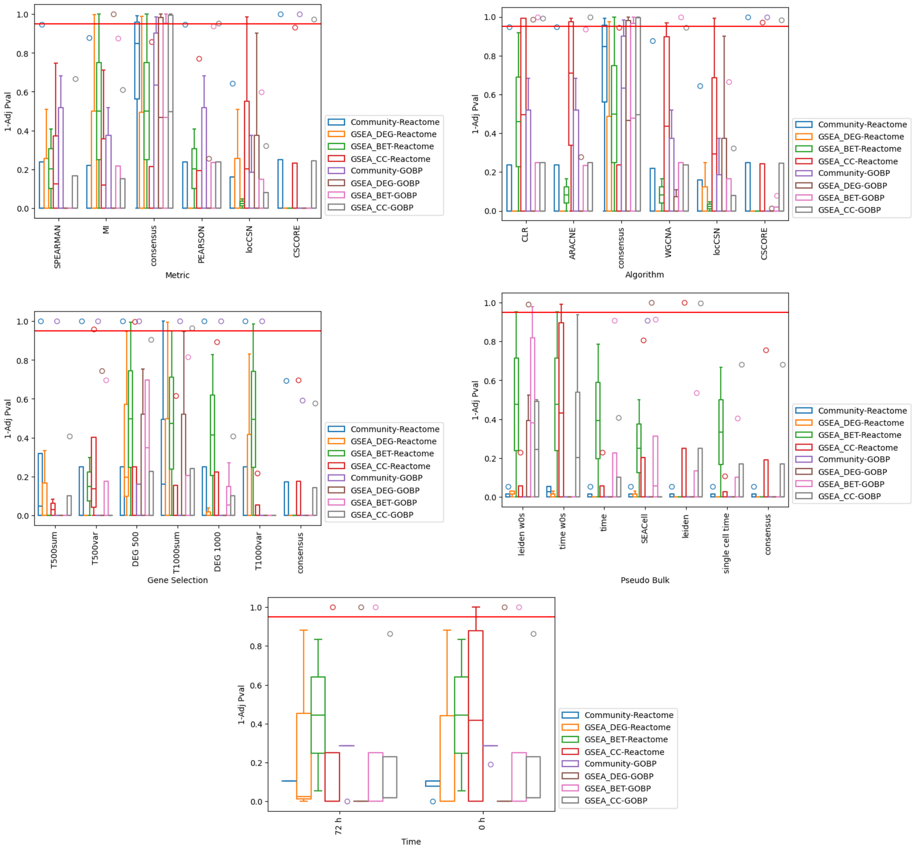

Figure 20: 1- adjusted p-value distribution of gene co-expression network cluster enrichment for gene- and community-based enrichment. Clusters are based on the similarity of the assigned Reactome and GOBP terms of the individual networks. The red horizontal line indicates 0.95. GSEA: (node-based analysis, based on node centralities; DEG (degree centrality), BET (betweenness centrality), CC (closeness centrality)). Community: (community-based network analysis).

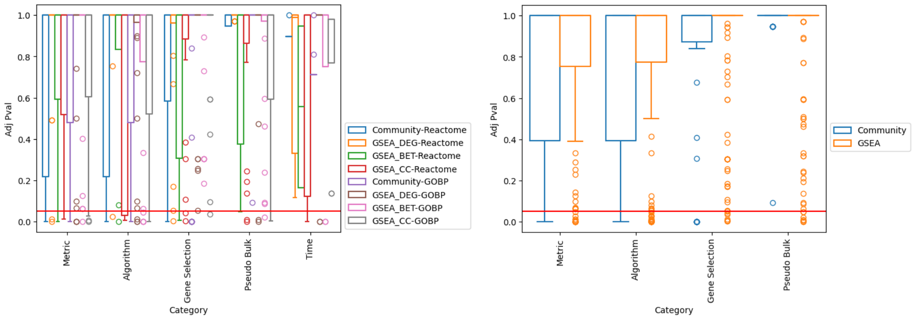

Figure 21: Box plot of parameter categories that are over-represented in clusters grouped by analysis method. If a parameter is over-represented in a cluster, this implies that the parameter influences that clustering, which may suggest a (technical) bias. Clusters are based on the similarity of the assigned Reactome and GOBP terms of the individual networks. Left: Reactome and GOBP are separated, node-based (GSEA) and community-based analyses are also separated. Right: Reactome and GOBP are grouped together, all node-based (GSEA) analyses are also grouped together. GSEA: (node-based analysis, based on node centralities; DEG (degree centrality), BET (betweenness centrality), CC (closeness centrality)). Community: (community-based network analysis).

7.3.3 Rosa et al.

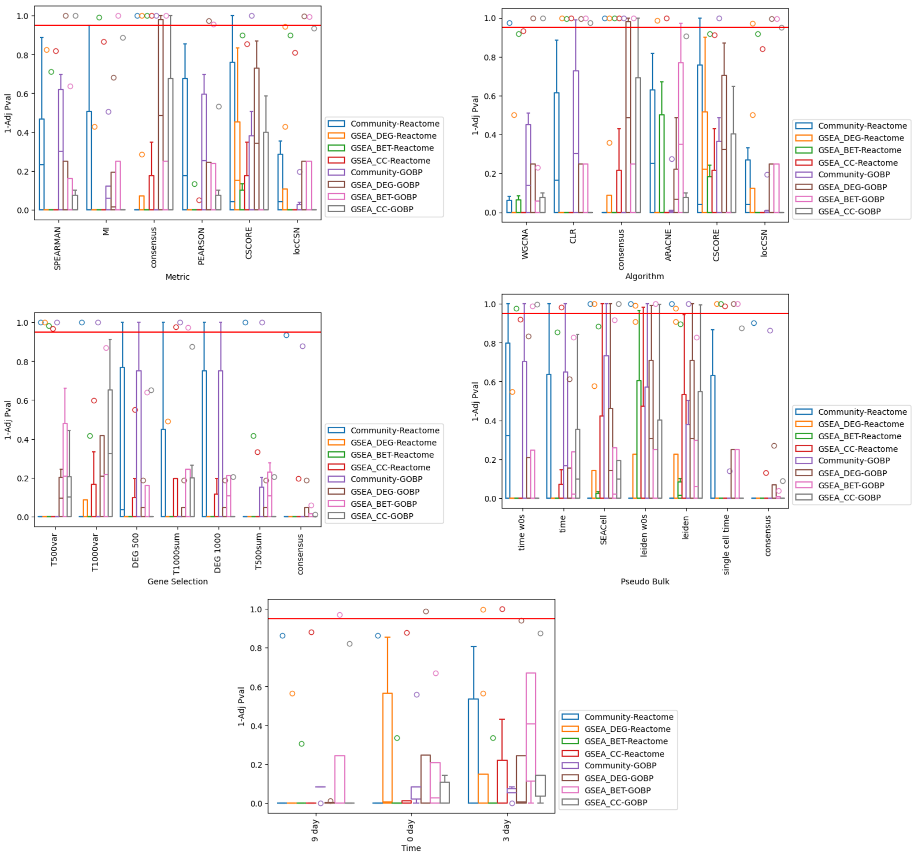

Figure 22: 1- adjusted p-value distribution of gene co-expression network cluster enrichment for gene- and community-based enrichment. Clusters are based on the similarity of the assigned Reactome and GOBP terms of the individual networks. The red horizontal line indicates 0.95. GSEA: (node-based analysis, based on node centralities; DEG (degree centrality), BET (betweenness centrality), CC (closeness centrality)). Community: (community-based network analysis).

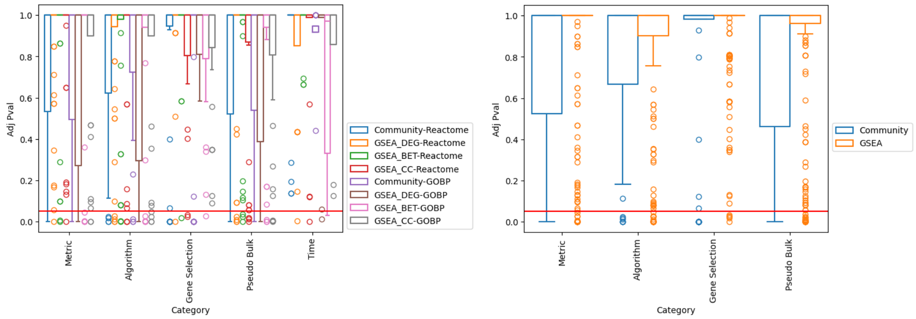

Figure 23: Box plot of parameter categories that are over-represented in clusters grouped by analysis method. If a parameter is over-represented in a cluster, this implies that the parameter influences that clustering, which may suggest a (technical) bias. Clusters are based on the similarity of the assigned Reactome and GOBP terms of the individual networks. Left: Reactome and GOBP are separated, node-based (GSEA) and community-based analyses are also separated. Right: Reactome and GOBP are grouped together, all node-based (GSEA) analyses are also grouped together. GSEA: (node-based analysis, based on node centralities; DEG (degree centrality), BET (betweenness centrality), CC (closeness centrality)). Community: (community-based network analysis).

7.3.4 Close et al.

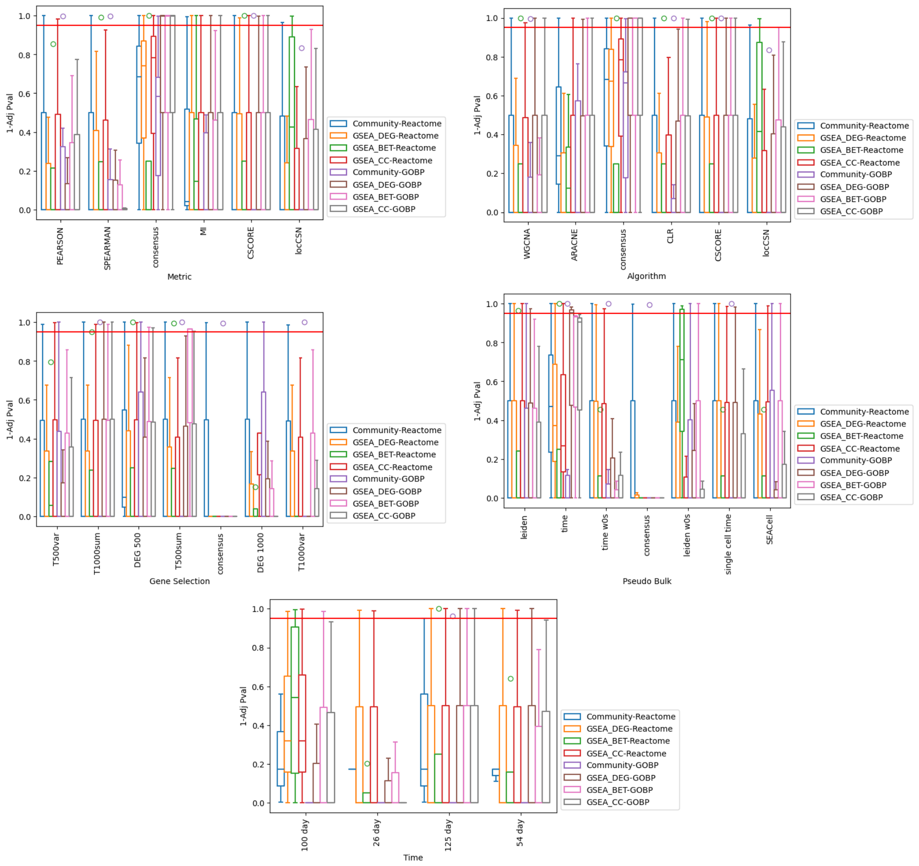

Figure 24: 1- adjusted p-value distribution of gene co-expression network cluster enrichment for gene- and community-based enrichment. Clusters are based on the similarity of the assigned Reactome and GOBP terms of the individual networks. The red horizontal line indicates 0.95. GSEA: (node-based analysis, based on node centralities; DEG (degree centrality), BET (betweenness centrality), CC (closeness centrality)). Community: (community-based network analysis).

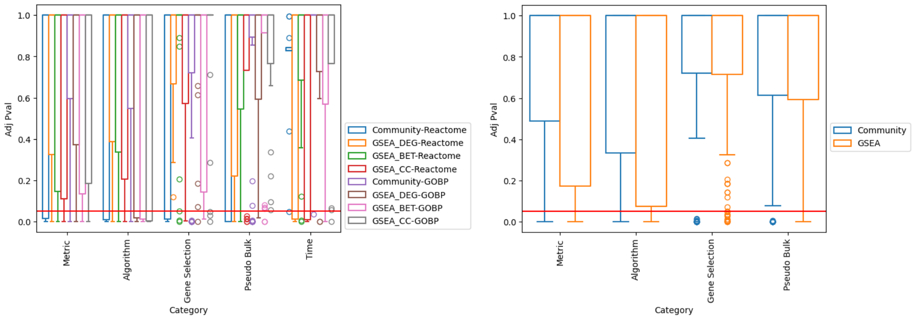

Figure 25: Box plot of parameter categories that are over-represented in clusters grouped by analysis method. If a parameter is over-represented in a cluster, this implies that the parameter influences that clustering, which may suggest a (technical) bias. Clusters are based on the similarity of the assigned Reactome and GOBP terms of the individual networks. Left: Reactome and GOBP are separated, node-based (GSEA) and community-based analyses are also separated. Right: Reactome and GOBP are grouped together, all node-based (GSEA) analyses are also grouped together. GSEA: (node-based analysis, based on node centralities; DEG (degree centrality), BET (betweenness centrality), CC (closeness centrality)). Community: (community-based network analysis).

8 Results vs. PubMed

8.1 Combined and Single Time Point Networks

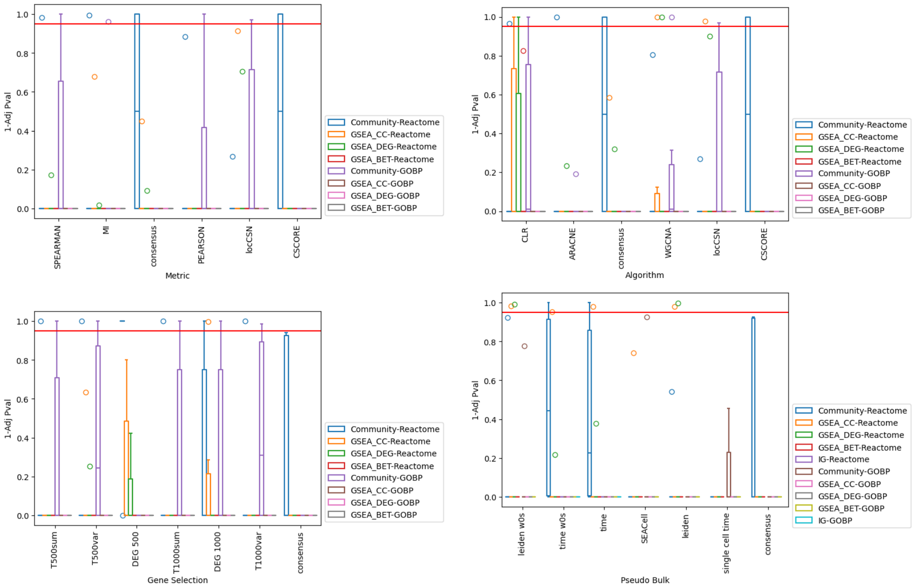

Figure 26: Box plots of the adjusted p-value distribution of gene co-expression network cluster enrichment (over-representation) for gene- and community-based enrichment on the cluster containing the expected (prior) results. Clusters are based on the PubMed scores for the enriched GOBP and Reactome terms. The red horizontal line indicates 0.95. GSEA: (node-based analysis, based on node centralities; DEG (degree centrality), BET (betweenness centrality), CC (closeness centrality)). Community: (community-based network analysis).

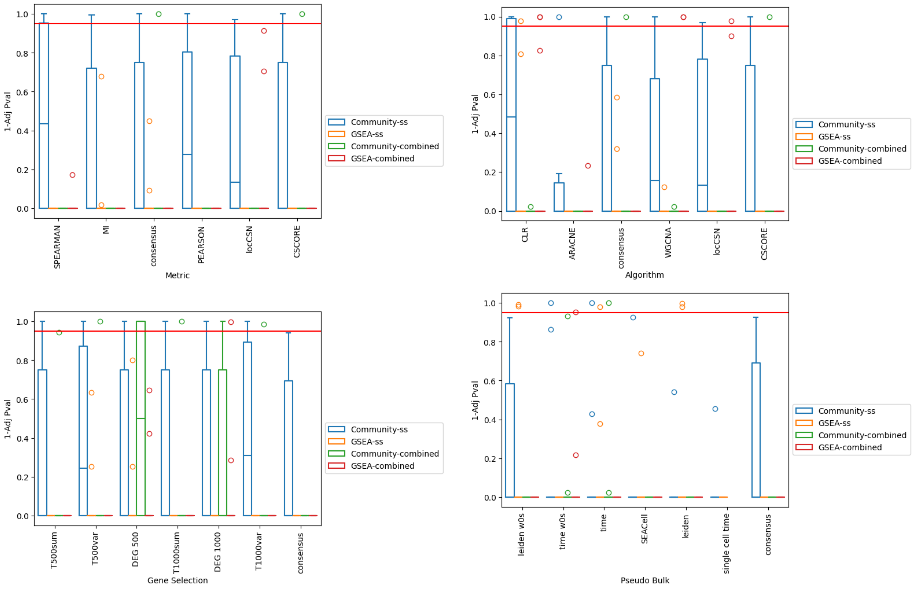

Figure 27: Box plots of the adjusted p-value distribution of gene co-expression network cluster enrichment (over-representation) for gene- and community-based enrichment on the cluster containing the expected (prior) results grouped by analysis method. Clusters are based on the PubMed scores for the enriched GOBP and Reactome terms. The red horizontal line indicates 0.95. GSEA: (node-based analysis). Community: (community-based network analysis). ss: single time point modeling. combined: combined time point modeling.

8.2 Combined Co-expression Network

8.2.1 All Datasets

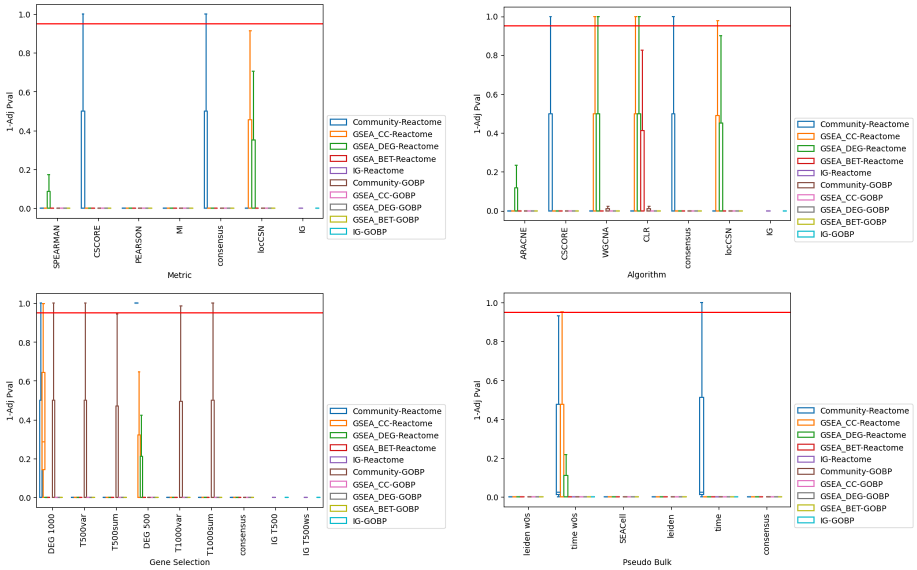

Figure 28: Box plots of the adjusted p-value distribution of gene co-expression network cluster enrichment (over-representation) for gene- and community-based enrichment on the cluster containing the expected (prior) results. Clusters are based on the PubMed scores for the enriched GOBP and Reactome terms. The red horizontal line indicates 0.95. GSEA: (node-based analysis, based on node centralities; DEG (degree centrality), BET (betweenness centrality), CC (closeness centrality)). Community: (community-based network analysis).

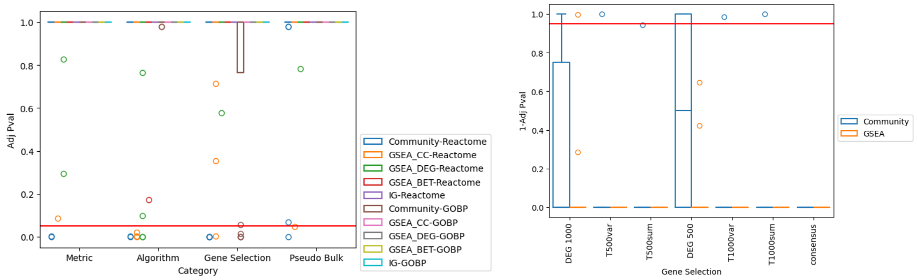

Figure 29: Box plots of the adjusted p-value distribution of gene co-expression network cluster enrichment (over-representation) for gene- and community-based enrichment on the cluster containing the expected (prior) results grouped by analysis method. Clusters are based on the PubMed scores for the enriched GOBP and Reactome terms. The red horizontal line indicates 0.95. GSEA: (node-based analysis). Community: (community-based network analysis). ss: single time point modeling. combined: combined time point modeling.

8.2.2 Yiangou et al.

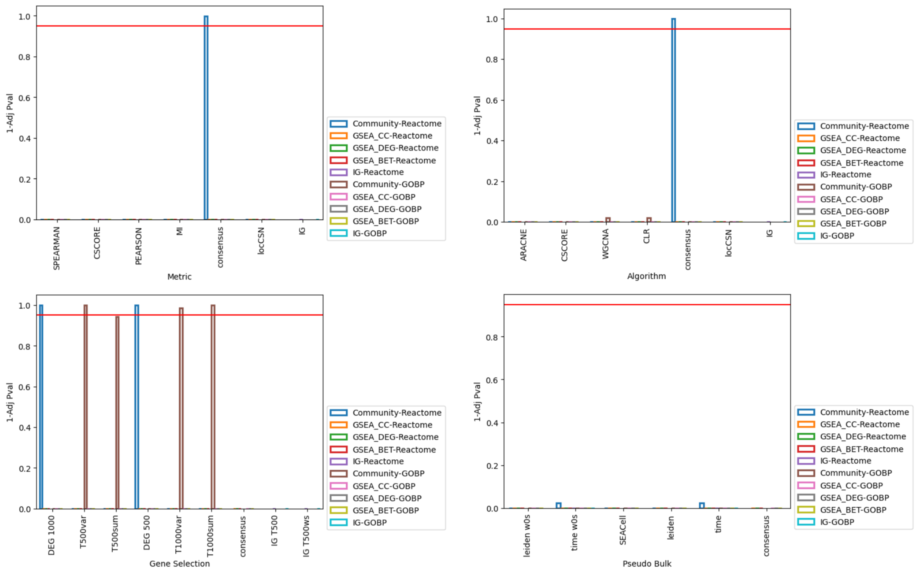

Figure 30: Box plots of the adjusted p-value distribution of gene co-expression network cluster enrichment (over-representation) for gene- and community-based enrichment on the cluster containing the expected (prior) results. Clusters are based on the PubMed scores for the enriched GOBP and Reactome terms. The red horizontal line indicates 0.95. GSEA: (node-based analysis, based on node centralities; DEG (degree centrality), BET (betweenness centrality), CC (closeness centrality)). Community: (community-based network analysis).

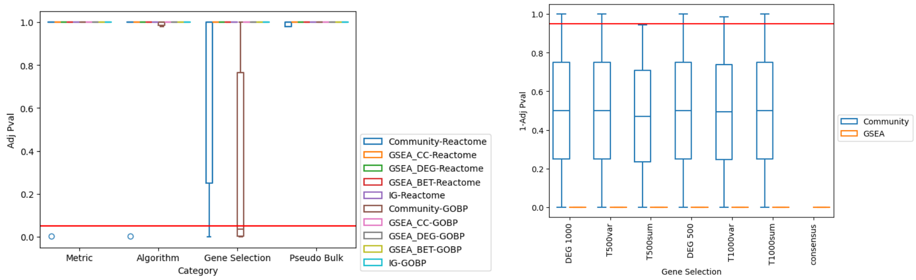

Figure 31: Box plots of the adjusted p-value distribution of gene co-expression network cluster enrichment (over-representation) for gene- and community-based enrichment on the cluster containing the expected (prior) results grouped by analysis method. Clusters are based on the PubMed scores for the enriched GOBP and Reactome terms. The red horizontal line indicates 0.95. GSEA: (node-based analysis). Community: (community-based network analysis). ss: single time point modeling. combined: combined time point modeling.

8.2.3 Rosa et al.

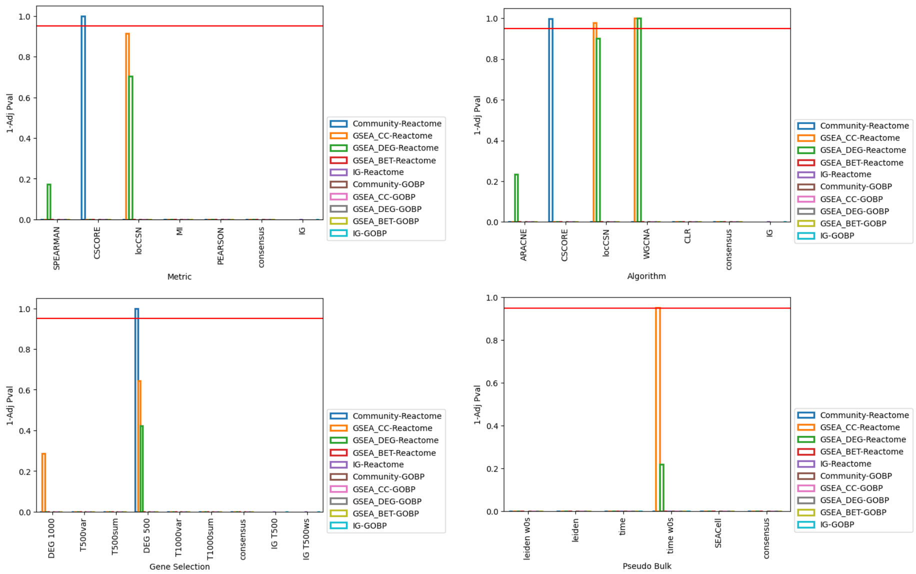

Figure 32: Box plots of the adjusted p-value distribution of gene co-expression network cluster enrichment (over-representation) for gene- and community-based enrichment on the cluster containing the expected (prior) results. Clusters are based on the PubMed scores for the enriched GOBP and Reactome terms. The red horizontal line indicates 0.95. GSEA: (node-based analysis, based on node centralities; DEG (degree centrality), BET (betweenness centrality), CC (closeness centrality)). Community: (community-based network analysis).

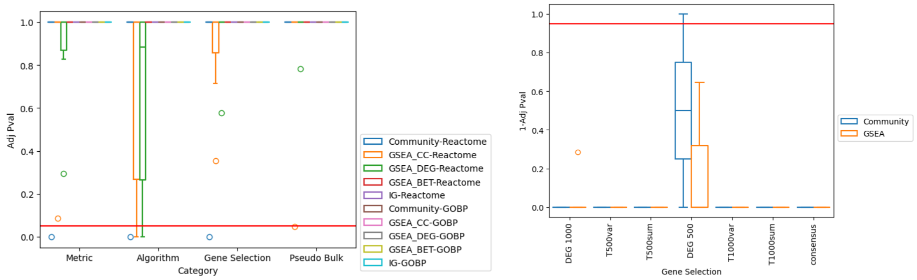

Figure 33: Box plots of the adjusted p-value distribution of gene co-expression network cluster enrichment (over-representation) for gene- and community-based enrichment on the cluster containing the expected (prior) results grouped by analysis method. Clusters are based on the PubMed scores for the enriched GOBP and Reactome terms. The red horizontal line indicates 0.95. GSEA: (node-based analysis). Community: (community-based network analysis). ss: single time point modeling. combined: combined time point modeling.

8.2.4 Close et al.

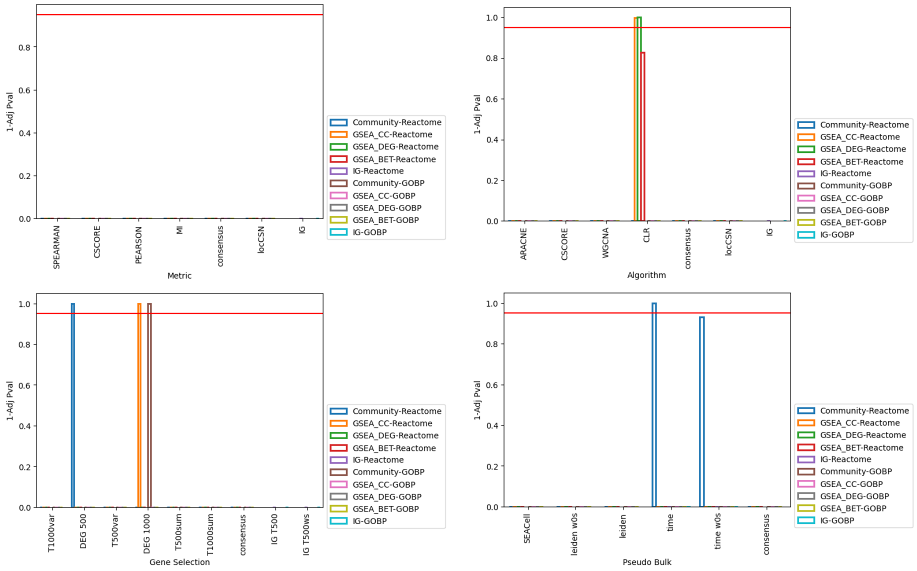

Figure 34: Box plots of the adjusted p-value distribution of gene co-expression network cluster enrichment (over-representation) for gene- and community-based enrichment on the cluster containing the expected (prior) results. Clusters are based on the PubMed scores for the enriched GOBP and Reactome terms. The red horizontal line indicates 0.95. GSEA: (node-based analysis, based on node centralities; DEG (degree centrality), BET (betweenness centrality), CC (closeness centrality)). Community: (community-based network analysis).

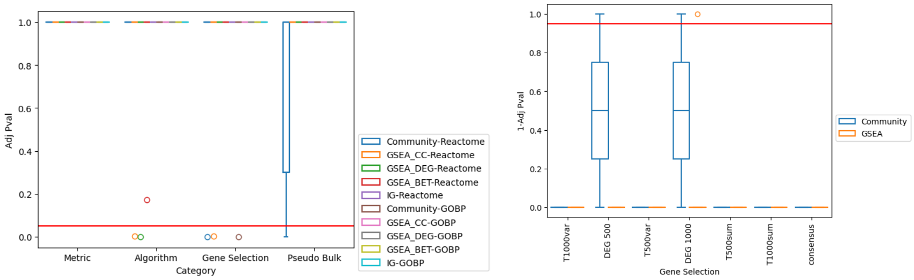

Figure 35: Box plots of the adjusted p-value distribution of gene co-expression network cluster enrichment (over-representation) for gene- and community-based enrichment on the cluster containing the expected (prior) results grouped by analysis method. Clusters are based on the PubMed scores for the enriched GOBP and Reactome terms. The red horizontal line indicates 0.95. GSEA: (node-based analysis). Community: (community-based network analysis). ss: single time point modeling. combined: combined time point modeling.

8.3 Single Time Point Co-expression Network

8.3.1 All Datasets

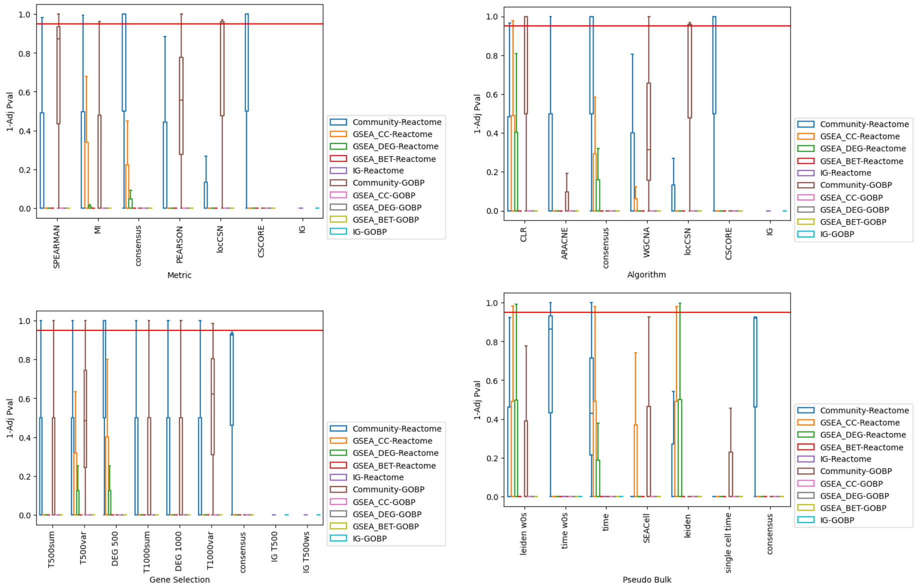

Figure 36: Box plots of the adjusted p-value distribution of gene co-expression network cluster enrichment (over-representation) for gene- and community-based enrichment on the cluster containing the expected (prior) results. Clusters are based on the PubMed scores for the enriched GOBP and Reactome terms. The red horizontal line indicates 0.95. GSEA: (node-based analysis, based on node centralities; DEG (degree centrality), BET (betweenness centrality), CC (closeness centrality)). Community: (community-based network analysis).

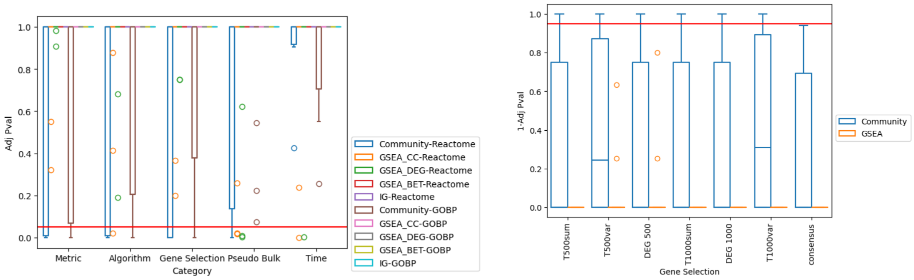

Figure 37: Box plots of the p-value distribution of gene co-expression network cluster enrichment (over-representation) for gene- and community-based enrichment on the cluster containing the expected (prior) results. Clusters are based on the PubMed scores for the enriched GOBP and Reactome terms. The red horizontal lines indicate 0.05 and 0.95, respectively. Left: Reactome and GOBP are separated. Right: Reactome and GOBP are grouped together. GSEA: (node-based analysis, based on node centralities; DEG (degree centrality), BET (betweenness centrality), CC (closeness centrality)). Community: (community-based network analysis).

8.3.2 Yiangou et al.

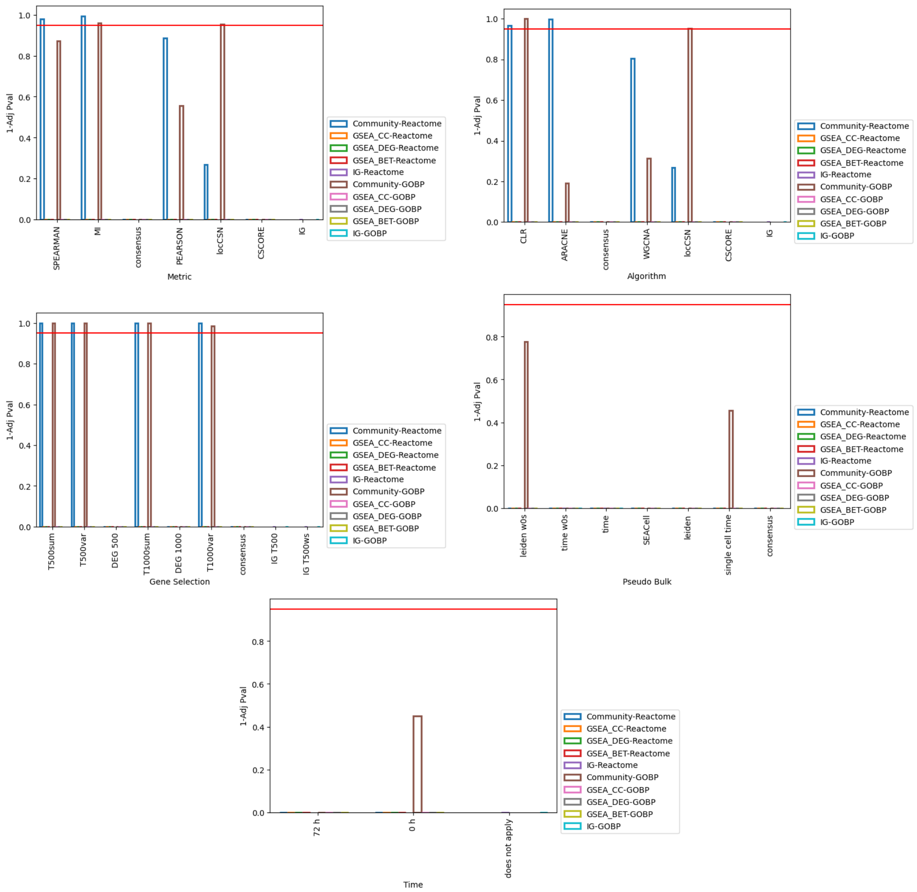

Figure 38: Box plots of the adjusted p-value distribution of gene co-expression network cluster enrichment (over-representation) for gene- and community-based enrichment on the cluster containing the expected (prior) results. Clusters are based on the PubMed scores for the enriched GOBP and Reactome terms. The red horizontal line indicates 0.95. GSEA: (node-based analysis, based on node centralities; DEG (degree centrality), BET (betweenness centrality), CC (closeness centrality)). Community: (community-based network analysis).

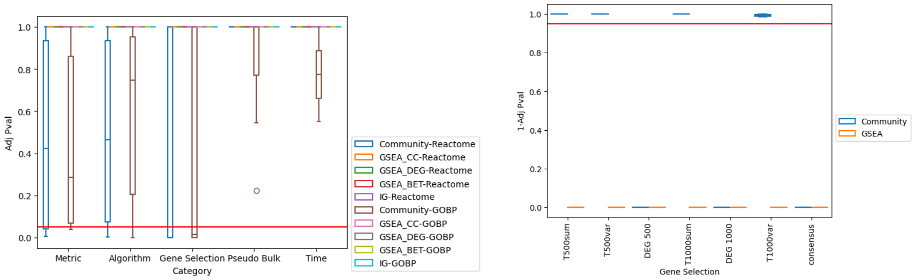

Figure 39: Box plots of the p-value distribution of gene co-expression network cluster enrichment (over-representation) for gene- and community-based enrichment on the cluster containing the expected (prior) results. Clusters are based on the PubMed scores for the enriched GOBP and Reactome terms. The red horizontal lines indicate 0.05 and 0.95, respectively. Left: Reactome and GOBP are separated. Right: Reactome and GOBP are grouped together. GSEA: (node-based analysis, based on node centralities; DEG (degree centrality), BET (betweenness centrality), CC (closeness centrality)). Community: (community-based network analysis).

8.4 Rosa et al.

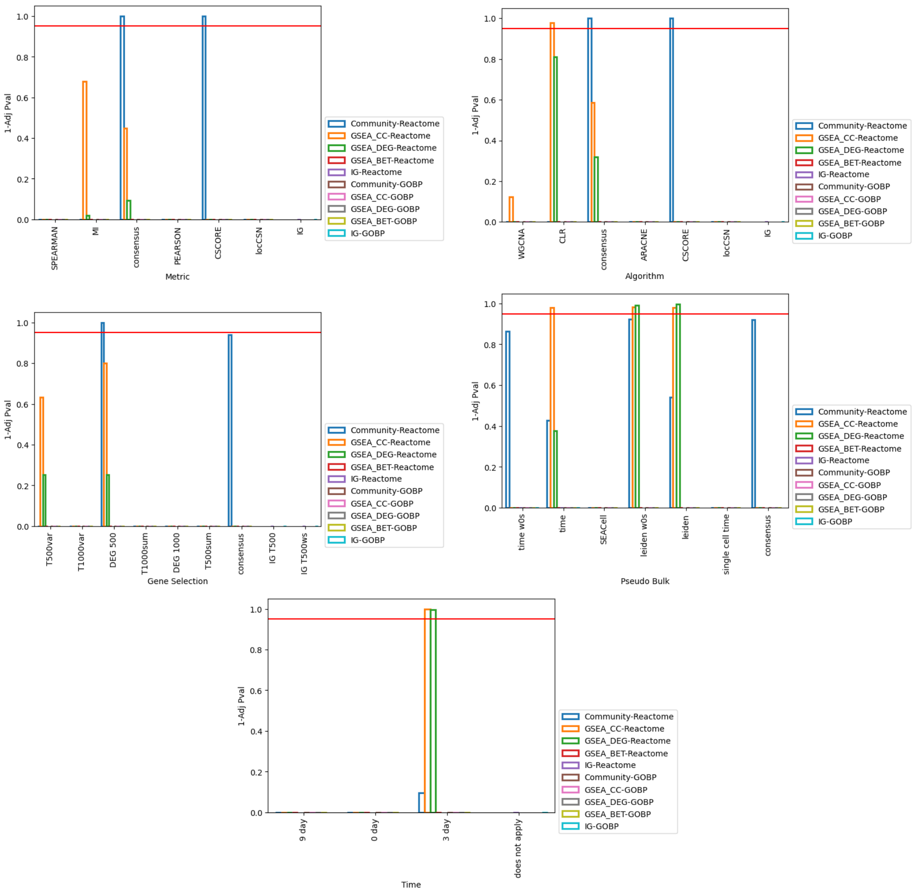

Figure 40: Box plots of the adjusted p-value distribution of gene co-expression network cluster enrichment (over-representation) for gene- and community-based enrichment on the cluster containing the expected (prior) results. Clusters are based on the PubMed scores for the enriched GOBP and Reactome terms. The red horizontal line indicates 0.95. GSEA: (node-based analysis, based on node centralities; DEG (degree centrality), BET (betweenness centrality), CC (closeness centrality)). Community: (community-based network analysis).

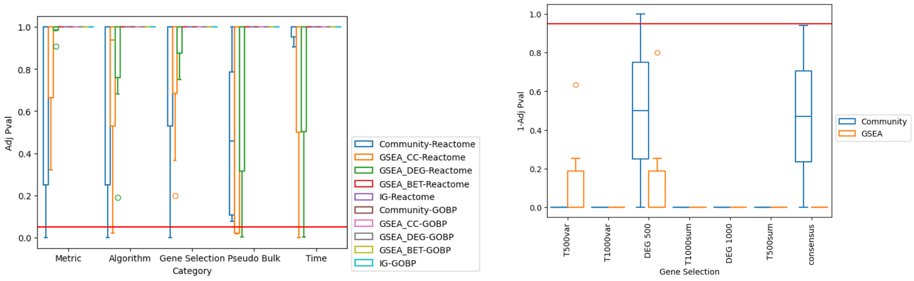

Figure 41: Box plots of the p-value distribution of gene co-expression network cluster enrichment (over-representation) for gene- and community-based enrichment on the cluster containing the expected (prior) results. Clusters are based on the PubMed scores for the enriched GOBP and Reactome terms. The red horizontal lines indicate 0.05 and 0.95, respectively. Left: Reactome and GOBP are separated. Right: Reactome and GOBP are grouped together. GSEA: (node-based analysis, based on node centralities; DEG (degree centrality), BET (betweenness centrality), CC (closeness centrality)). Community: (community-based network analysis).

8.5 Close et al.

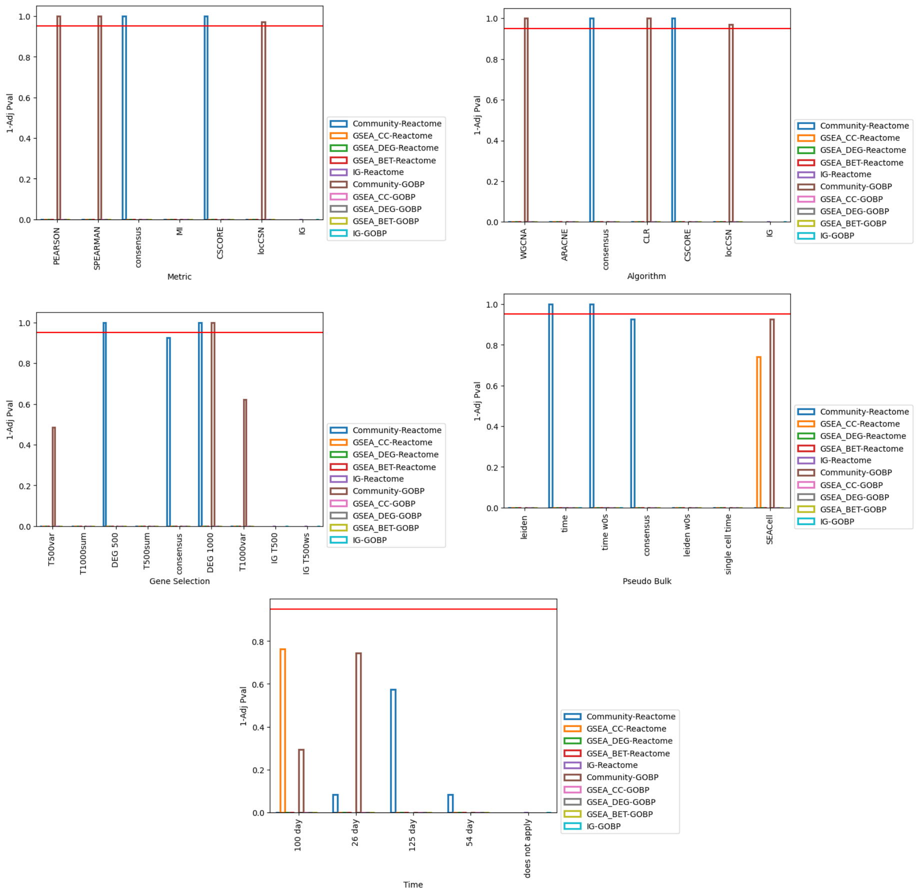

Figure 42: Box plots of the adjusted p-value distribution of gene co-expression network cluster enrichment (over-representation) for gene- and community-based enrichment on the cluster containing the expected (prior) results. Clusters are based on the PubMed scores for the enriched GOBP and Reactome terms. The red horizontal line indicates 0.95. GSEA: (node-based analysis, based on node centralities; DEG (degree centrality), BET (betweenness centrality), CC (closeness centrality)). Community: (community-based network analysis).

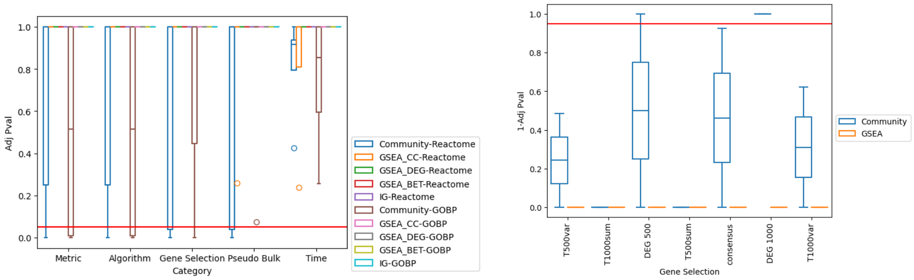

Figure 43: Box plots of the p-value distribution of gene co-expression network cluster enrichment (over-representation) for gene- and community-based enrichment on the cluster containing the expected (prior) results. Clusters are based on the PubMed scores for the enriched GOBP and Reactome terms. The red horizontal lines indicate 0.05 and 0.95, respectively. Left: Reactome and GOBP are separated. Right: Reactome and GOBP are grouped together. GSEA: (node-based analysis, based on node centralities; DEG (degree centrality), BET (betweenness centrality), CC (closeness centrality)). Community: (community-based network analysis).

9 Similarity of Analysis Methods and their Results

9.1 Yiangou et al.

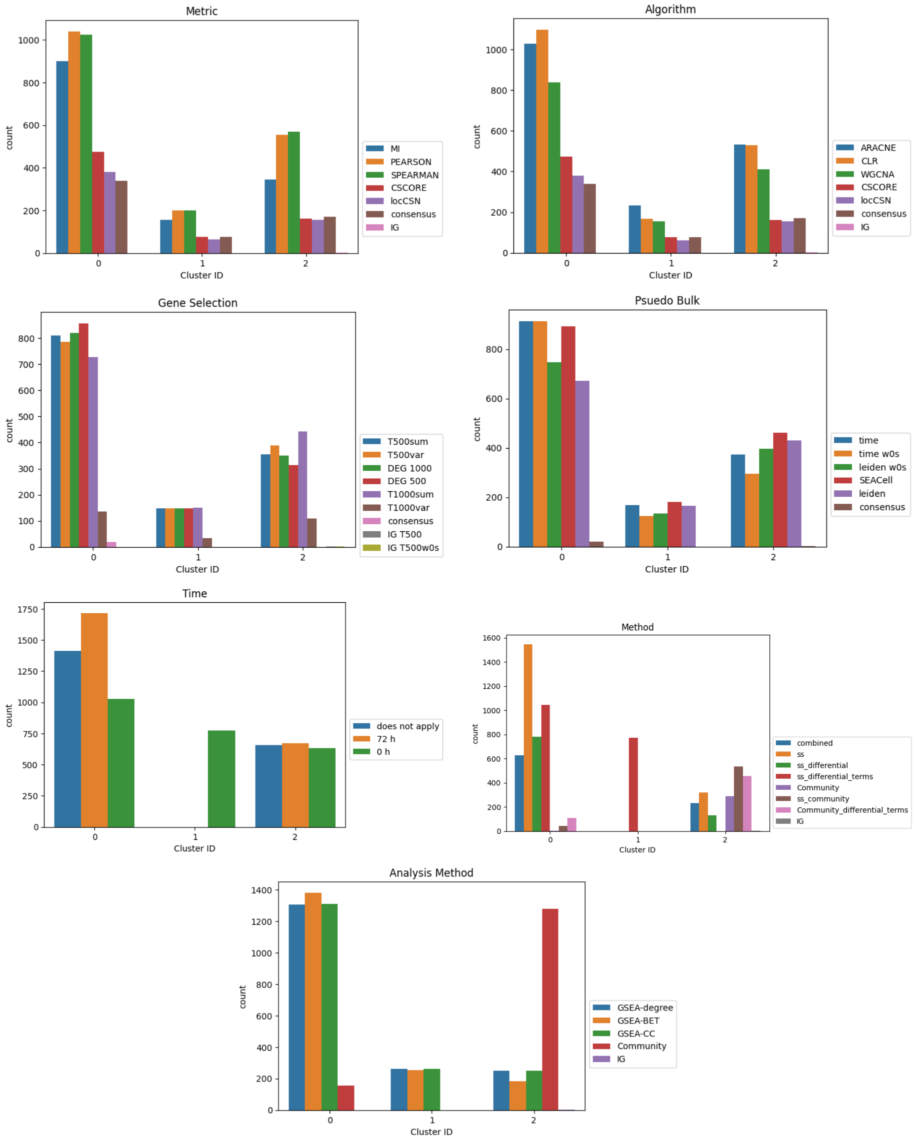

Figure 44: Count plot of parameters falling into different clusters based on the Jaccard distance between the resulting Reactome terms for each analysis method.

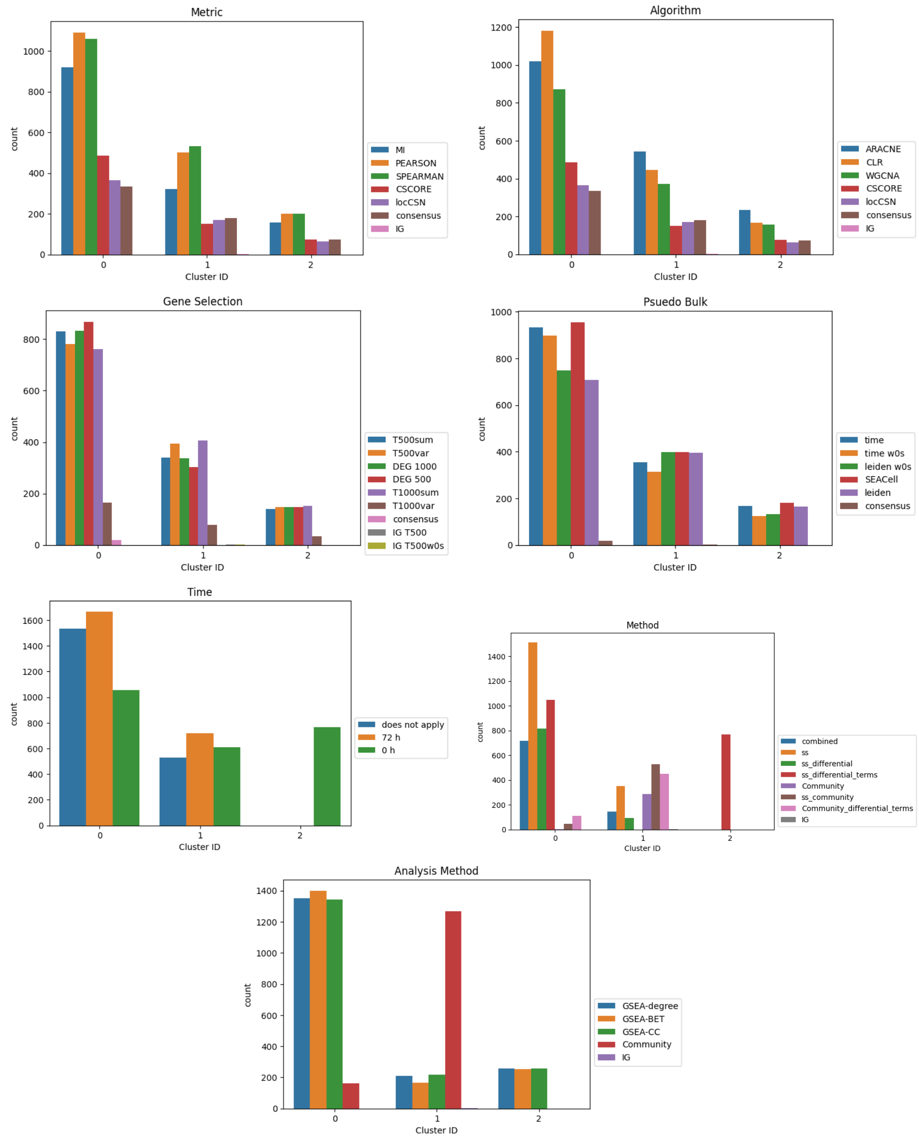

Figure 45: Count plot of parameters falling into different clusters based on the Jaccard distance between the resulting GOBP terms for each analysis method.

9.2 Rosa et al.

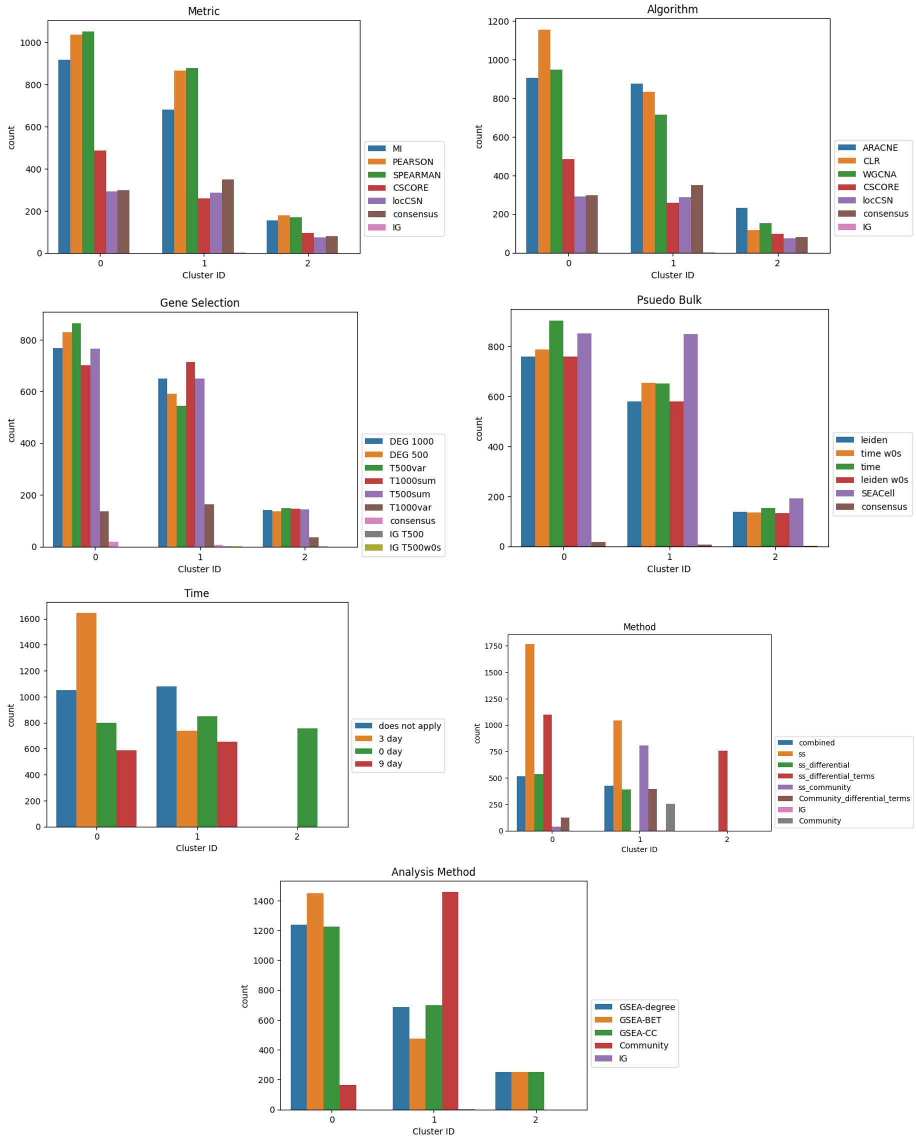

Figure 46: Count plot of parameters falling into different clusters based on the Jaccard distance between the resulting Reactome terms for each analysis method.

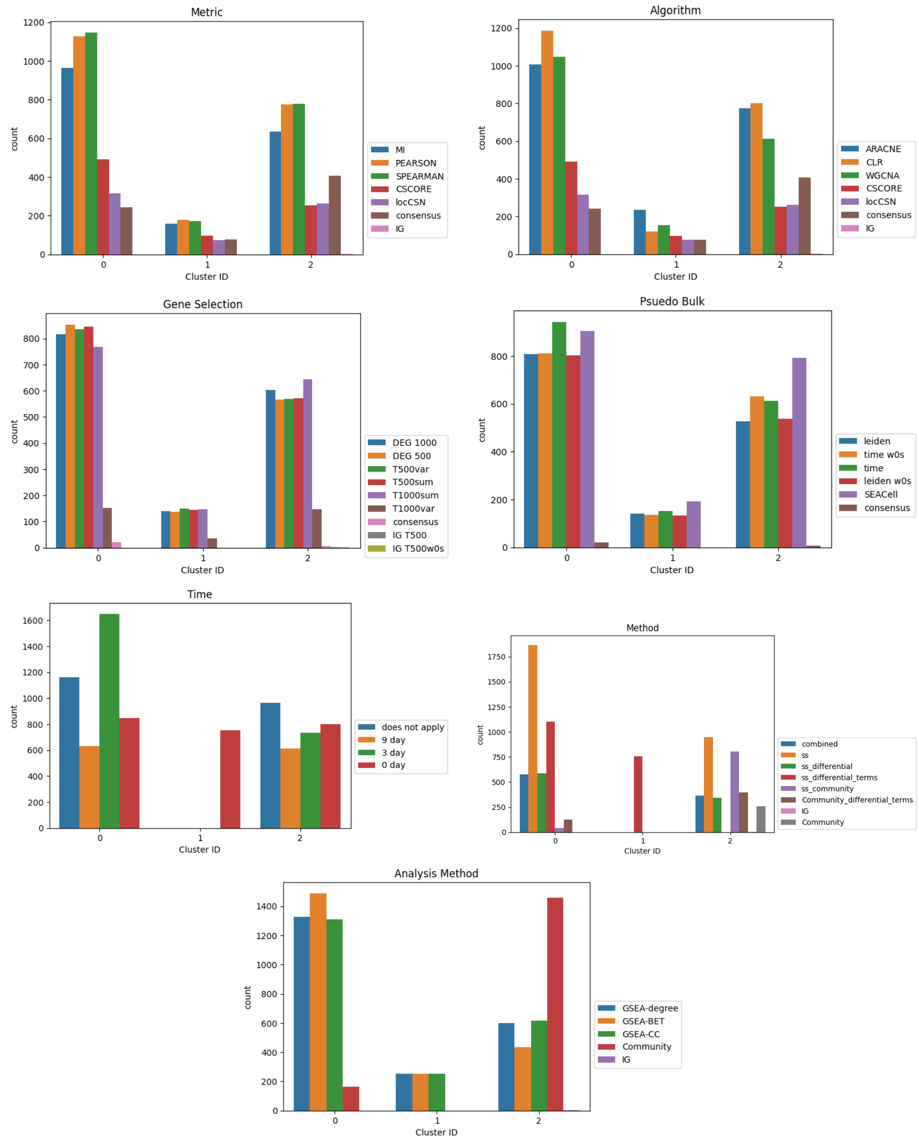

Figure 47: Count plot of parameters falling into different clusters based on the Jaccard distance between the resulting GOBP terms for each analysis method.

9.3 Close et al.

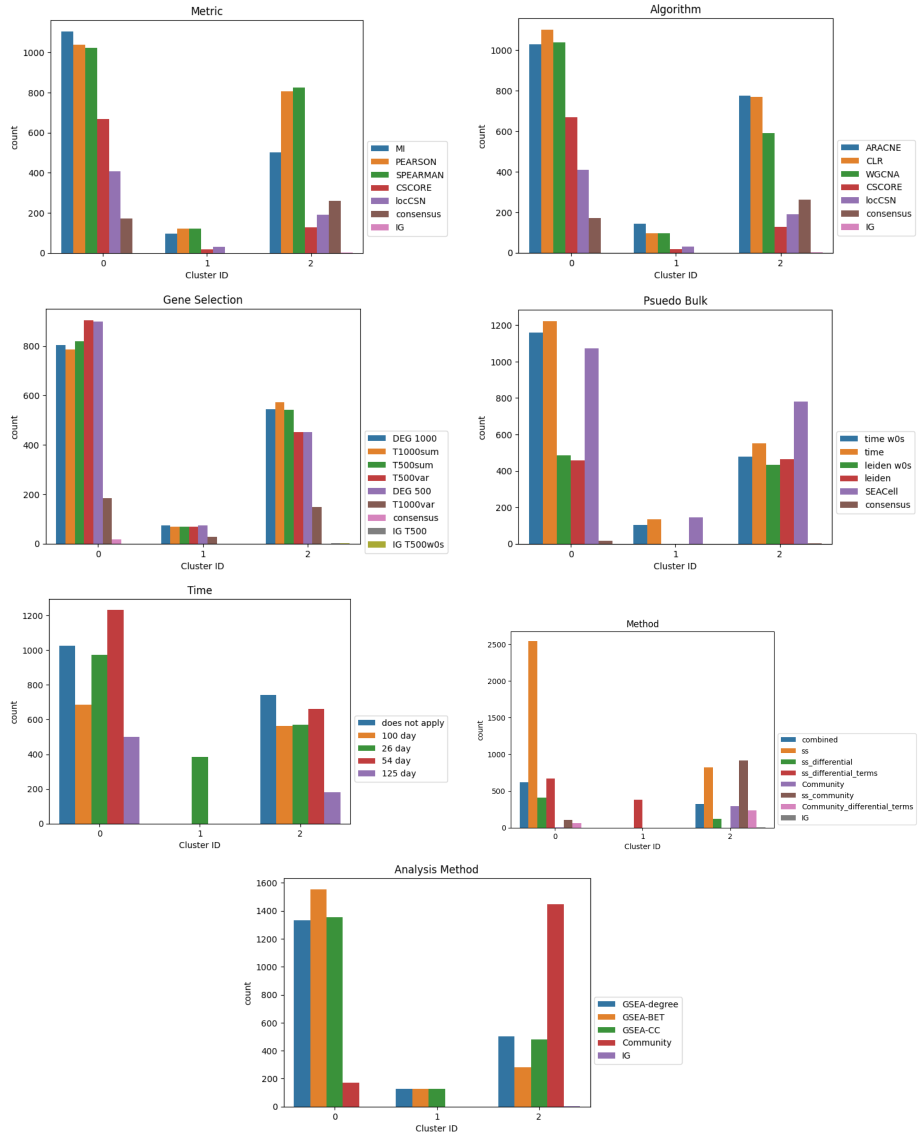

Figure 48: Count plot of parameters falling into different clusters based on the Jaccard distance between the resulting Reactome terms for each analysis method.

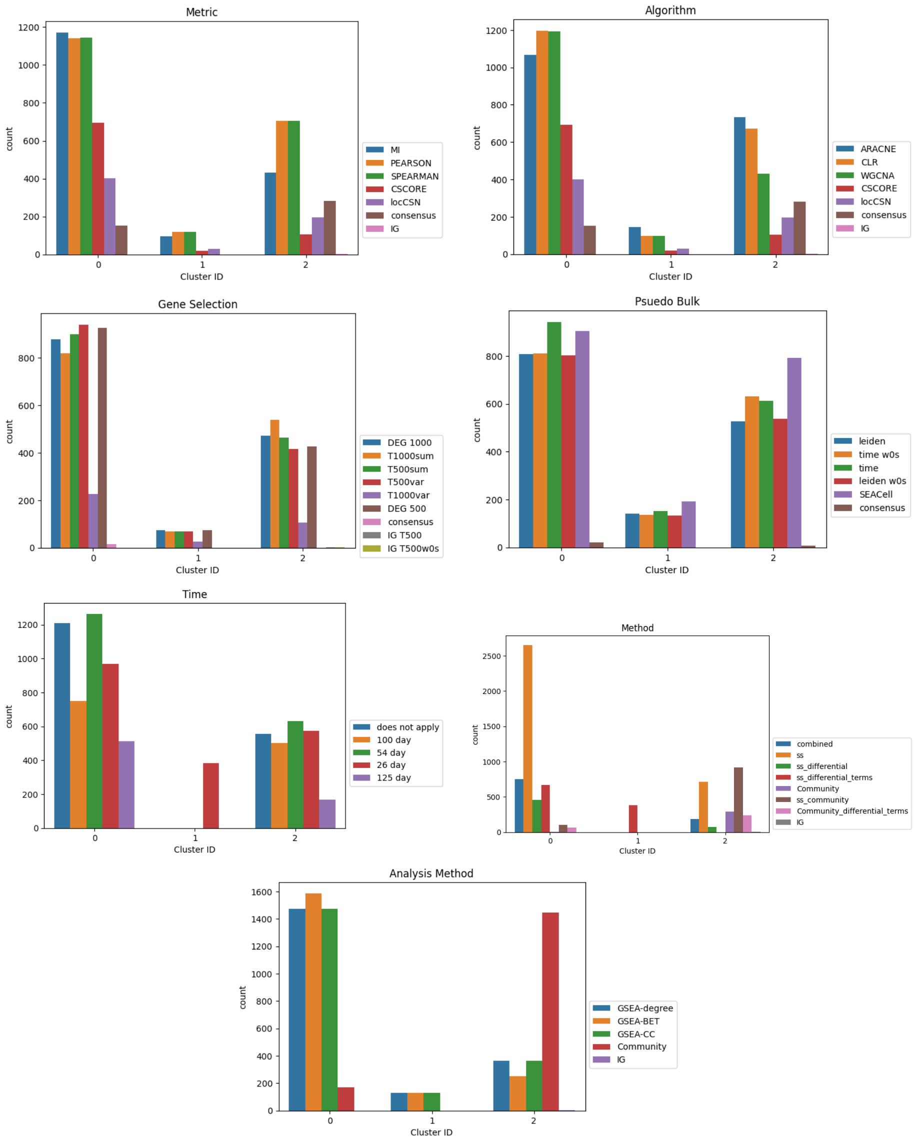

Figure 49: Count plot of parameters falling into different clusters based on the Jaccard distance between the resulting GOBP terms for each analysis method.

References

[1] F Alexander Wolf, Philipp Angerer, and Fabian J Theis. Scanpy: large-scale single-cell gene expression data analysis. *Genome biology*, 19:1–5, 2018.

[2] Adam A Margolin, Ilya Nemenman, Katia Basso, Chris Wiggins, Gustavo Stolovitzky, Riccardo Dalla Favera, and Andrea Califano. Aracne: an algorithm for the reconstruction of gene regulatory networks in a mammalian cellular context. In *BMC bioinformatics*, volume 7, pages 1–15. Springer, 2006.

[3] Patrick E Meyer, Frederic Lafitte, and Gianluca Bontempi. minet: Ar/bioconductor package for inferring large transcriptional networks using mutual information. *BMC bioinformatics*, 9:1–10, 2008.

[4] Jeremiah J Faith, Boris Hayete, Joshua T Thaden, Ilaria Mogno, Jamey Wierzbowski, Guillaume Cottarel, Simon Kasif, James J Collins, and Timothy S Gardner. Large-scale mapping and validation of escherichia coli transcriptional regulation from a compendium of expression profiles. *PLoS biology*, 5(1):e8, 2007.

[5] Chang Su, Zichun Xu, Xinning Shan, Biao Cai, Hongyu Zhao, and Jingfei Zhang. Cell-type-specific co-expression inference from single cell rna-sequencing data. *Nature Communications*, 14(1):4846, 2023.

[6] Xuran Wang, David Choi, and Kathryn Roeder. Constructing local cell-specific networks from single-cell data. *Proceedings of the National Academy of Sciences*, 118(51):e2113178118, 2021.

[7] Peter Langfelder and Steve Horvath. Wgcna: an r package for weighted correlation network analysis. *BMC bioinformatics*, 9:1–13, 2008.

[8] Narges Rezaie, Farilie Reese, and Ali Mortazavi. Pywgcna: a python package for weighted gene co-expression network analysis. *Bioinformatics*, 39(7):btad415, 2023.

[9] The pandas development team. pandas-dev/pandas: Pandas, February 2020.

[10] Wes McKinney. Data Structures for Statistical Computing in Python. In Stéfan van der Walt and Jarrod Millman, editors, *Proceedings of the 9th Python in Science Conference*, pages 56 – 61, 2010.

[11] Samuel Morabito, Fairlie Reese, Negin Rahimzadeh, Emily Miyoshi, and Vivek Swarup. hdwgcna identifies co-expression networks in high-dimensional transcriptomics data. *Cell reports methods*, 3(6), 2023.

[12] Veer Singh Marwah, Pia Anneli Sofia Kinaret, Angela Serra, Giovanni Scala, Antti Lauerma, Vittorio Fortino, and Dario Greco. Inform: inference of network response modules. *Bioinformatics*, 34(12):2136–2138, 2018.

[13] Gregorio Alanis-Lobato, Miguel A Andrade-Navarro, and Martin H Schaefer. Hippie v2. 0: enhancing meaningfulness and reliability of protein–protein interaction networks. *Nucleic acids research*, page gkw985, 2016.

[14] Aric Hagberg, Pieter J Swart, and Daniel A Schult. Exploring network structure, dynamics, and function using networkx. Technical report, Los Alamos National Laboratory (LANL), Los Alamos, NM (United States), 2008.

[15] Pauli Virtanen, Ralf Gommers, Travis E. Oliphant, Matt Haberland, Tyler Reddy, David Cournapeau, Evgeni Burovski, Pearu Peterson, Warren Weckesser, Jonathan Bright, Stéfan J. van der Walt, Matthew Brett, Joshua Wilson, K. Jarrod Millman, Nikolay Mayorov, Andrew R. J. Nelson, Eric Jones, Robert Kern, Eric Larson, C J Carey, İlhan Polat, Yu Feng, Eric W. Moore, Jake VanderPlas, Denis Laxalde, Josef Perktold, Robert Cimrman, Ian Henriksen, E. A. Quintero, Charles R. Harris, Anne M. Archibald, Antônio H. Ribeiro, Fabian Pedregosa, Paul van Mulbregt, and SciPy 1.0 Contributors. SciPy 1.0: Fundamental Algorithms for Scientific Computing in Python. *Nature Methods*, 17:261–272, 2020.

[16] Skipper Seabold and Josef Perktold. statsmodels: Econometric and statistical modeling with python. In *9th Python in Science Conference*, 2010.

[17] Yoav Benjamini and Yosef Hochberg. Controlling the false discovery rate: a practical and powerful approach to multiple testing. *Journal of the Royal statistical society: series B (Methodological)*, 57(1):289–300, 1995.

[18] Zhuoqing Fang, Xinyuan Liu, and Gary Peltz. Gseapy: a comprehensive package for performing gene set enrichment analysis in python. *Bioinformatics*, 39(1):btac757, 2023.

[19] Marc Gillespie, Bijay Jassal, Ralf Stephan, Marija Milacic, Karen Rothfels, Andrea Senff-Ribeiro, Johannes Griss, Cristoffer Sevilla, Lisa Matthews, Chuqiao Gong, et al. The reactome pathway knowledgebase 2022. *Nucleic acids research*, 50(D1):D687–D692, 2022.

[20] Michael Ashburner, Catherine A Ball, Judith A Blake, David Botstein, Heather Butler, J Michael Cherry, Allan P Davis, Kara Dolinski, Selina S Dwight, Janan T Eppig, et al. Gene ontology: tool for the unification of biology. *Nature genetics*, 25(1):25–29, 2000.

[21] Suzi A Aleksander, James Balhoff, Seth Carbon, J Michael Cherry, Harold J Drabkin, Dustin Ebert, Marc Feuermann, Pascale Gaudet, Nomi L Harris, et al. The gene ontology knowledgebase in 2023. *Genetics*, 224(1):iyad031, 2023.

[22] Aravind Subramanian, Pablo Tamayo, Vamsi K Mootha, Sayan Mukherjee, Benjamin L Ebert, Michael A Gillette, Amanda Paulovich, Scott L Pomeroy, Todd R Golub, Eric S Lander, et al. Gene set enrichment analysis: a knowledge-based approach for interpreting genome-wide expression profiles. *Proceedings of the National Academy of Sciences*, 102(43):15545–15550, 2005.

[23] Alexander Lachmann, Zhuorui Xie, and Avi Ma'ayan. blitzgsea: efficient computation of gene set enrichment analysis through gamma distribution approximation. *Bioinformatics*, 38(8):2356–2357, 2022.

[24] Alisa Pavel, Antonio Federico, Giusy Del Giudice, Angela Serra, and Dario Greco. Volta: advanced molecular network analysis. *Bioinformatics*, 37(23):4587–4588, 2021.

[25] Edward Y Chen, Christopher M Tan, Yan Kou, Qiaonan Duan, Zichen Wang, Gabriela Vaz Meirelles, Neil R Clark, and Avi Ma'ayan. Enrichr: interactive and collaborative html5 gene list enrichment analysis tool. *BMC bioinformatics*, 14:1–14, 2013.

[26] Maxim V Kuleshov, Matthew R Jones, Andrew D Rouillard, Nicolas F Fernandez, Qiaonan Duan, Zichen Wang, Simon Koplev, Sherry L Jenkins, Kathleen M Jagodnik, Alexander Lachmann, et al. Enrichr: a comprehensive gene set enrichment analysis web server 2016 update. *Nucleic acids research*, 44(W1):W90–W97, 2016.

[27] Zhuorui Xie, Allison Bailey, Maxim V Kuleshov, Daniel JB Clarke, John E Evangelista, Sherry L Jenkins, Alexander Lachmann, Megan L Wojciechowicz, Eryk Kropiwnicki, Kathleen M Jagodnik, et al. Gene set knowledge discovery with enrichr. *Current protocols*, 1(3):e90, 2021.

[28] Vincent A Traag, Ludo Waltman, and Nees Jan Van Eck. From louvain to leiden: guaranteeing well-connected communities. *Scientific reports*, 9(1):1–12, 2019.

[29] Michael L. Waskom. seaborn: statistical data visualization. *Journal of Open Source Software*, 6(60):3021, 2021.

[30] Eric W Sayers, Evan E Bolton, J Rodney Brister, Kathi Canese, Jessica Chan, Donald C Comeau, Ryan Connor, Kathryn Funk, Chris Kelly, Sunghwan Kim, et al. Database resources of the national center for biotechnology information. *Nucleic acids research*, 50(D1):D20–D26, 2022.

[31] F. Pedregosa, G. Varoquaux, A. Gramfort, V. Michel, B. Thirion, O. Grisel, M. Blondel, P. Prettenhofer, R. Weiss, V. Dubourg, J. Vanderplas, A. Passos, D. Cournapeau, M. Brucher, M. Perrot, and E. Duchesnay. Scikit-learn: Machine learning in Python. *Journal of Machine Learning Research*, 12:2825–2830, 2011.
